# Precision and Accuracy of Radiological Bone Age Assessment in Children among Different Ethnic Groups: A Systematic Review

**DOI:** 10.3390/diagnostics13193124

**Published:** 2023-10-04

**Authors:** Sebastián Eustaquio Martín Pérez, Isidro Miguel Martín Pérez, Jesús María Vega González, Ruth Molina Suárez, Coromoto León Hernández, Fidel Rodríguez Hernández, Mario Herrera Perez

**Affiliations:** 1 Departamento de Farmacología y Medicina Física, Área de Radiología y Medicina Física, Sección de Enfermería y Fisioterapia, Facultad de Ciencias de la Salud, Universidad de La Laguna, 38200 Santa Cruz de Tenerife, Spain; alu0100713374@ull.edu.es (I.M.M.P.); frguezh@ull.edu.es (F.R.H.); 2 Escuela de Doctorado y Estudios de Posgrado, Universidad de La Laguna, San Cristóbal de La Laguna, 38203 Santa Cruz de Tenerife, Spain; 3 Musculoskeletal Pain and Motor Control Research Group, Faculty of Health Sciences, Universidad Europea de Canarias, 38300 Santa Cruz de Tenerife, Spain; 4 Musculoskeletal Pain and Motor Control Research Group, Faculty of Sport Sciences, Universidad Europea de Madrid, 28670 Villaviciosa de Odón, Spain; 5 Institute of Legal Medicine and Forensic Sciences of Santa Cruz de Tenerife, 38230 San Cristóbal de La Laguna, Spain; jveggonf@justiciaencanarias.org; 6 Pediatric Endocrinology Unit, Pediatric Department, Hospital Universitario de Canarias, San Cristóbal de La Laguna, 38320 Santa Cruz de Tenerife, Spain; rmolsua@gobiernodecanarias.org; 7 Departamento de Ingeniería Informática y de Sistemas, Universidad de La Laguna, Apdo. 456, San Cristóbal de La Laguna, 38200 Santa Cruz de Tenerife, España; cleon@ull.es; 8 School of Medicine (Health Sciences), Universidad de La Laguna, 38200 Santa Cruz de Tenerife, Spain; mherrera@ull.es; 9 Foot and Ankle Unit, Orthopedic Surgery and Traumatology Department, San Cristóbal de La Laguna, 38320 Santa Cruz de Tenerife, Spain

**Keywords:** diagnostic imaging, radiography, age determination by skeleton, racial group, children

## Abstract

Aim: The aim was to identify, evaluate, and summarize the findings of relevant individual studies on the precision and accuracy of radiological BA assessment procedures among children from different ethnic groups. Materials and Methods: A qualitative systematic review was carried out following the MOOSE statement and previously registered in *PROSPERO* (CRD42023449512). A search was performed in MEDLINE (*PubMed*) (*n* = 561), the Cochrane Library (*n* = 261), CINAHL (*n* = 103), Web of Science (*WOS*) (*n* = 181), and institutional repositories (*n* = 37) using MeSH and free terms combined with the Booleans “*AND*” and “*OR*”. *NOS* and *ROBINS-E* were used to assess the methodological quality and the risk of bias of the included studies, respectively. Results: A total of 51 articles (*n* = 20,100) on radiological BA assessment procedures were precise in terms of intra-observer and inter-observer reliability for all ethnic groups. In Caucasian and Hispanic children, the Greulich–Pyle Atlas (*GPA*) was accurate at all ages, but in youths, Tanner–Whitehouse radius–ulna–short bones 3 (*TW3-RUS*) could be an alternative. In Asian and Arab subjects, GPA and Tanner–Whitehouse 3 (*TW3*) overestimated the BA in adolescents near adulthood. In African youths, GPA overestimated the BA while TW3 was more accurate. Conclusion: GPA and TW3 radiological BA assessment procedures are both precise but their accuracy in estimating CA among children of different ethnic groups can be altered by racial bias.

## 1. Introduction

Radiological methods of determining bone age (BA) are the process of estimating chronological age (CA) by observing radiographic markers in the skeletal bones [1,2,3] of the pediatric population [4,5,6]. Classical methods of radiological BA determination are based on the detection of changes in morphological appearance by comparison with a reference atlas of left anteroposterior hand–wrist radiographs such as the Greulich–Pyle Atlas (*GPA*) or Tanner–Whitehouse (*TW*) [7] method, or panoramic dental X-ray, such as Demirjian or FELS [6,8,9,10,11,12].

With respect to radiological skeletal methods, GPA is the most widely used method of estimating BA in medical practice. This method is based on the shape and maturity of primary and secondary ossification centers, and the timing of fusion between them [13,14,15]. However, the TW method consists of evaluating regions of interest (ROI) in specific bones of the left hand and classifying them in grades from A to I. An updated version of the TW3 method has added new radius, ulna, and short bone imaging regions [16]. This process assigns a score to each bone segment evaluated and is more detailed than a simple comparison with a standard radiography such as GPA. Gender differences were also considered, which is important in pediatric patients.

Regarding radiological dental methods, Demirjian is the most widely used dental method for determining BA and is based on the evaluation of seven mandibular teeth on the left side according to the eight-stage dental development system [17,18]. The individual values obtained for each tooth are added to obtain an overall maturity value from which the tooth age is determined using an age conversion table. The FELS method involves detecting maturity indicators as radiographic features of the wrist, thereby determining the maturity index and metric maturity index. The method was developed using radiographs of children through a standardized selection procedure to identify useful indices, with the radius, ulna, and carpal bones contributing more to the final value than others [19,20].

Despite its applicability and simplicity, BA assessment is complex even for experts [9] because skeletal maturity is not uniform and appears to depend on both non-modifiable factors, such as genetic factors, and modifiable factors, such as diet or environmental living conditions [4,21]. Furthermore, previous studies have shown differences in markers of skeletal maturity among ethnic groups, and thus racial differences require the development of new radiographic age determination methods [22,23,24].

Alternatively, some authors such as Eklof and Ringertz developed a method to assess maturity based on bone length and width in Scandinavian children [25], Schmid and Moll developed criteria for white Germans [26], and, in order to avoid racial differences in determining BA, the Sugiura Nakazawa method published standards for male and female Japanese children [27]. Willems developed a method to assess BA aimed at reducing the influence of ethnic and environmental factors [28]. Other methods performed on North African children showed significant differences between estimated BA and CA [29].

Given the above, potential racial bias in these radiology procedures may alter their precision and accuracy based on the racial group to which children belong [4]. Systematic use of standard radiological methods for BA assessment may lead to incorrect decisions by experts, such as pediatricians caring for children with advanced or delayed growth, or forensic estimation of CA among migrant children [30,31]. Therefore, it is essential for experts to obtain up-to-date and reliable information on the metric properties of radiological methods used for BA assessment, not only to resolve medical but also ethical and legal issues.

Furthermore, the lack of prior studies on this research question calls for a comprehensive study with a systematic approach that will help to obtain information on the suitability of these diagnostic methods for the radiographic determination of CA among the main ethnicities. As a consequence, the aim was to identify, evaluate, and summarize the findings of relevant individual studies on the precision and accuracy of radiological BA assessment procedures among children from different ethnic groups.

## 2. Materials and Methods

### 2.1. Study Design

A systematic review study was carried out from 1 June 2023 to 30 September 2023, with the defined protocol and was subdivided into four phases based on the standards of the *MOOSE* statement (Meta-analysis of Observational Studies in Epidemiology guidelines for meta-analyses and systematic reviews of observational studies) [32].

The protocol for this systematic review was previously registered in the International Prospective Registry of Systematic Reviews PROSPERO (CRD42023449512) and is available for consultation through the following website: https://www.crd.york.ac.uk/prospero/display_record.php?ID=CRD42023449512.

### 2.2. Search Strategy

A literature search was conducted from 1 August 2023 to 28 August 2023 to identify all available studies on the precision and accuracy of BA determination by skeletal or dental radiological diagnostic methods in the *MEDLINE (PubMed)*, *Cochrane Library*, *CINAHL,* and *Web of Science (WOS)* databases and other public institutional repositories.

In MEDLINE, the first search string was: “Reproducibility of results” [Mesh] OR “Dimensional Measurements Accuracy” [Mesh] OR “Diagnostic Techniques and Procedures” [Mesh] OR “Diagnostic imaging” [Mesh] OR “Radiography” [Mesh] OR “Age Determination by Skeleton” [Mesh] OR “Bone matrix” [Mesh] OR “Carpal bones” [Mesh] OR “radius” [Mesh] OR “Racial Groups” [Mesh] OR “Race factors” [Mesh] OR “White people” [Mesh] OR “Black people” [Mesh] OR “Hispanic or Latino” [Mesh] OR “Asian people” [Mesh] OR “Native Hawaiian or Other Pacific Islander”[Mesh] OR “American Indian or Alaska Native”[Mesh] OR “Pacific Island People”[Mesh] OR “Asian American Native Hawaiian and Pacific Islander”[Mesh] OR “Bone Maturity” [tw] “Skeletal Maturation” [tw] OR “Skeletal Age” [tw] OR “Age Measurement” [tw] OR radiograp * [tw] OR radiol * [tw].

Moreover, the second search string was: “Reproducibility of results” [Mesh] OR “Dimensional Measurements Accuracy” [Mesh] OR “Diagnostic Techniques and Procedures” [Mesh] OR “Diagnostic imaging” [Mesh] OR “Radiography” [Mesh] OR “Radiography, panoramic” [Mesh] OR “Age Determination by Teeth” [Mesh] OR “Dentition” [Mesh] OR “Teeth” [Mesh] OR “Tooth” [Mesh] OR “Molar, Third” [Mesh] OR “Incisor” [Mesh] OR “Racial Groups” [Mesh] OR “Race factors” [Mesh] OR “White people” [Mesh] OR “Black people” [Mesh] OR “Hispanic or Latino” [Mesh] OR “Asian people” [Mesh] “Native Hawaiian or Other Pacific Islander”[Mesh] OR “American Indian or Alaska Native”[Mesh] OR “Pacific Island People”[Mesh] OR “Asian American Native Hawaiian and Pacific Islander”[Mesh] OR “BA measurement” [tw] OR “Orthopantomography” [tw] OR “Bone Maturity” [tw] “Skeletal Maturation” [tw] OR “Skeletal Age” [tw] OR “Age Measurement” [tw] OR radiograp*[tw] OR radiol * [tw].

Similar search strings were used in the *Cochrane Library*, *CINAHL*, and *Web of Science (WOS)* databases and public institutional repositories. Two independent researchers (SMP and IMP) performed the search and a blinded researcher, MHP, scored all the retrieved articles by title and abstract and then scored full-text publications to determine their eligibility. In case of discrepancies, a fourth author served as the decision judge (FHR). The search strategy is shown in Table 1.

### 2.3. Selection and Data Extraction

The selection criteria were: (1) observational studies (cohorts and cross-sectional), case reports, classical articles, clinical conferences, comments, comparative studies, evaluation studies, congress proceedings, consensus development conferences, dictionaries, editorials, letters, government publications, guidelines, historical articles, lectures, legal cases, legislation (2) published in English, Spanish, French, and Portuguese (3) recruiting children (6 to 12 yrs), adolescents (13 to 18 yrs), and young adults (19 to 24 yrs) (4) of any ethnic group (5) undergoing BA determination by skeletal, dental, or cervical radiography procedures (6) published in MEDLINE (*PubMed*), Cochrane Library, CINAHL, and Web of Science *(WOS)* databases and public institutional repositories (7) available in full-text and (8) that have measured at least outcomes related to the precision or accuracy or measurements related to radiological BA assessment.

Data extraction was performed independently by two authors (IMP and SMP), and in case of disagreement, a third author (FHR) was responsible for resolving disagreements. A standardized work template based on the PECO question was used to extract and detail all information about the authors, year and country of publication, study design, outcomes, participants (sample size, gender, type of radiological projection, institutional information, etc.), radiographic method, and results of measured outcomes.

The *Cochrane Handbook for Systematic Reviews of Interventions-v.5.1.0* was used to develop these sections. The reliability of the table was tested using a representative sample of the studies to be reviewed.

### 2.4. Methodological Quality Assessment

Non-randomized clinical trials or observational studies were assessed using the *Newcastle Ottawa Scale (NOS)* [33]. This analysis tool is based on several domains including the selection of the group study (*4 points*), the compatibility between the data (*2 points*), and the interpretation of results (*3 points*). For the evaluation of a study using NOS, each of the 7 questions asked received a star rating in the sample selection and evaluation of results, with a maximum of two stars for compatibility, corresponding to a maximum score of 9 points.

### 2.5. Risk of Bias Assessment

Risk of bias analyses of observational studies and trials were independently performed by MHP using the *Cochrane Risk of Bias Tool for observational studies of exposures (ROBINS-E)* [34]. This assessment instrument includes flagging questions that should be addressed within each confounding domain, the selection of study participants, classification of exposures, deviations from expected exposures, missing data, outcome measurement, and selection of reported outcomes. The response options are: “*Low risk*”, “*Some concerns*”, “*High risk*”, “*Very risk*”, and “*No information*”. Based on the score obtained in the analysis of the domains of the tool, the existence of a low, some concern, high, and very high risk of bias was interpreted globally. Any disagreement between the authors was resolved by discussion, and in case of conflicting scores, the third reviewer (FRH) was called upon to make the decision.

## 3. Results

### 3.1. Study Selection

A total of 1143 studies were identified in the MEDLINE (*PubMed*) (*n* = 561), Cochrane Library (*n* = 261), CINAHL (*n* = 103), and Web of Science *(WOS)* (*n* = 181) databases, and public institutional repositories (*n* = 37). After eliminating duplicates (*n* = 671 articles), 472 studies were screened, eliminating a total of 393 after reading the title and abstract. Afterward, the full texts of the remaining 79 articles were evaluated, eliminating 28 because they did not match our previously established eligibility criteria.

**Figure 1:**
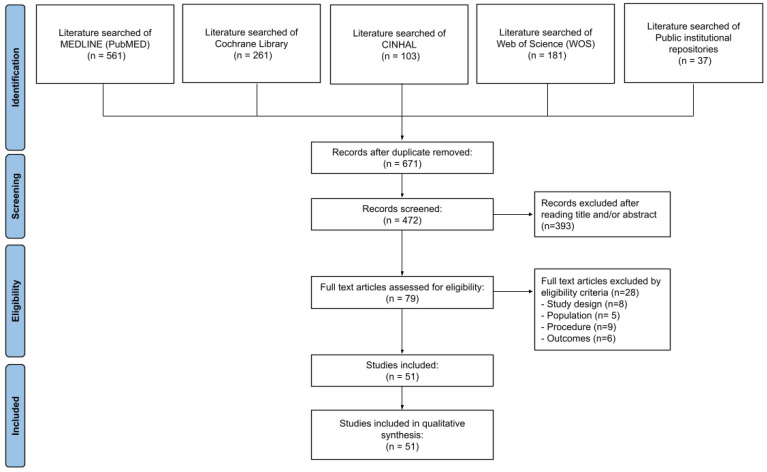
MOOSE flowchart of the selection process of observational studies.

A total of 88 papers were excluded because of different study designs, 5 were removed because they did not include subjects from the target populations, 9 papers were eliminated for not following the procedures for determining BA, and 6 studies were excluded for not measuring the required outcome variables. Finally, a total of *n* = 51 articles were included in the qualitative synthesis. The MOOSE flowchart of the selection process of observational studies is shown in Figure 1. 

### 3.2. Characteristics of Included Studies

The included studies were published between 1984 and 2023. Out of the 51 studies, 21 (41.18%) were conducted in different regions of Asia. Specifically, five studies were conducted in India [35,36,37,38,39], four in Turkey [40,41,42,43], three in Pakistan [30,44,45], two each in Saudi Arabia [46,47], China [48,49], and South Korea [50,51], and one each in Taiwan [52], Iran [53], and Israel [54].

In addition, 16 of the included studies were conducted in Europe, accounting for 31.37% of the total. Specifically, there were three studies in the United Kingdom [55,56,57], two in Spain [58,59], two in Portugal [60,61], two in Italy [62,63], two in France [64,65], one in Austria [66], one in Germany [67], one in the Netherlands [68,69], one in Sweden [69], and one conducted in Denmark [70].

Additionally, five studies (9.80%) were conducted exclusively in Africa, including two in South Africa [71,72], one in Zimbabwe [73] one in Botswana [74], and one in Ethiopia [75]. Also, a total of five studies (9.80%) were carried out in the region of Oceania. Specifically, two studies were conducted in Australia [76,77], one study was conducted in Malaysia [78], one study was conducted in Malaysia with radiographs of children from the United States [23], and one study was conducted in Thailand [79]. A total of four studies (7.84%) were run in the Americas, one in the United States of America [80], two in Venezuela [81,82], and one in Chile [83].

Regarding study design, 25 (49.02%) were observational retrospective cohort studies [23,35,40,42,43,48,50,52,54,55,57,58,61,62,63,64,65,66,67,68,69,76,79,83,84], followed by 19 (37.25%) cross-sectional studies [30,36,37,38,44,45,46,51,53,59,60,72,73,74,75,77,78,81,82], and 7 (13.73%) observational prospective cohort studies [39,41,55,56,70,71,80].

A total of 30 papers (58.8%) studied the accuracy [23,35,36,37,40,41,43,45,48,49,51,52,53,55,57,59,64,65,66,69,70,72,73,74,76,77,78,79,80,82], 1 of which also separately calculated the sensitivity and specificity [62]. A total of 27 (52.94%) studies analyzed precision [35,36,42,44,46,47,50,52,56,57,58,60,61,62,64,65,69,71,72,73,74,76,77,78,79,80,83], of which 23 (45.1%) assessed repeatability [35,42,44,46,47,50,52,56,57,58,60,61,62,64,69,71,72,73,74,77,78,79,80] and 18 (35.29%) evaluated reproducibility [36,42,44,46,47,50,52,58,60,61,62,65,73,74,76,78,79,83].

The studies included used postero-anterior projection radiographs of the hand and left wrist, antero-posterior panoramic orthopantomographies, and lateral cervical spine radiographs (*n* = 20,100) for BA assessment. The sample consisted of children between the ages of 0 [57,58,71] and 22 yrs [71].

The total number of studies conducted in Caucasian children was 23 (45.90%) representing a total of *n* = 9777 hand–wrist radiographs or panoramic radiography used to estimate BA in this ethnic group [23,40,41,43,54,55,56,57,58,59,61,62,63,64,65,66,67,68,70,76,77,79,80]. In particular, Spain accounts for a total of 1310 cases (13.39%) among Caucasians and approximately 6.51% of the total number of radiographs [59].

Secondly, the number of radiographs in studies conducted on Asian children was *n* = 3097 (15.40%). Of these studies, one subset focused specifically on Asian children (*n* = 2366, 11.77%) [23,47,48,49,50,51,52,80], while another subset focused on Indian (*n* = 731, 3.63%) [35,36,37,38,39] and Indonesian children [78,79].

Thirdly, a total of *n* = 4674 (*n* = 23.25%) radiographs were used to estimate BA in children of any Arab ethnicity [30,40,41,42,43,44,45,46,47,53,54]. Fourthly, the studies performed in Latin America, included 1728 radiographs, accounting for approximately 8.59% of the total sample [23,80,81,82,83]. Fifthly, the total number of studies carried out in African children was eight, representing *n* = 810 (4.02%) hand–wrist radiographs used to estimate the BA of this ethnic group [23,55,71,72,73,74,75,80]. Finally, 14 radiographies belonged to other ethnic groups such as Caucasian/Asiatic (*n* = 5) (0.02%) [55] and others (*n* = 9) (0.04%) [80].

Regarding the radiological skeletal methods, 92.2% of included studies used the manually applied Greulich and Pyle (GPA) method (*n* = 47) [23,35,36,37,39,40,41,43,44,45,46,47,48,49,50,51,52,53,54,55,56,57,58,59,60,61,62,63,64,65,66,67,68,69,70,71,72,73,74,75,76,77,78,79,80,81,83] followed by the Tanner–Whitehouse-3 method (*n* = 9) [38,47,48,49,50,51,55,62,73] and Tanner–Whitehouse-2 (*n* = 4) [56,59,62,70] Radiological dental procedures for BA assessment included Demirjian’s classification system [36,42,63,64,81], the FELS method [60], and the evaluation of cervical vertebra maturation (CVM), described by Mito et al. [38,42].

The included articles used other radiological techniques such as Girdany and Golden’s method [79], the Fishman method [48,79], the RUS-CHN approach [48], the McKay method [35,50], the Korean Standard BA method [50], the Thiemann and Nitz Atlas method [67], the Maturos method [42,61], and the hand and wrist maturation-Ru stage [42]. The characteristics of the included studies are presented in Table S1.

### 3.3. Methodological Quality Assessment (NOS)

The methodological quality assessment ranged from good to moderate with a mean of 6.2 (SD = 0.9) out of a total of 9 possible points. Content analysis showed that data availability (*n* = 48, 80.39%), the verification of the intervention (*n* = 35, 68.62%), and the evaluation of the result (*n* = 30,58.62%) were the domains of the scale that obtained the worst scores. Furthermore, 3 studies achieved the highest methodological quality, with a score of 8 out of 9 [45,73,78] while 11 studies achieved the lowest quality score with an overall of 5 out of 9 [30,38,49,56,59,63,65,68,75,76,83]. Details of the methodological quality assessment with the *NOS* are provided in Table 2. 

### 3.4. Risk of Bias Assessment (ROBINS-E)

The overall risk of bias assessed using the ROBINS-E instrument was high to very high. A total of 88.23% (*n* = 45) of studies [23,30,35,36,37,38,40,41,42,43,44,46,47,49,50,51,52,53,55,57,58,59,60,61,62,63,64,66,67,68,69,70,71,72,74,75,76,77,78,79,80,81,82,83] reported a high risk of bias of measurements of the exposure. In addition, 82.35% of the studies (*n* = 42) indicated a high selection risk of bias of the reported outcome [23,30,35,36,37,38,40,41,42,43,44,48,49,51,52,53,54,56,57,59,60,61,62,63,64,66,67,68,69,70,71,73,74,75,76,77,78,79,80,81,82,83]. Finally, the selection bias of the participants in the sample was high, accounting for 37.25% (*n* = 19) of the total articles included in this review [23,30,41,45,48,49,56,59,65,66,67,68,74,75,76,80,81,82,83]. The risk of bias analysis is detailed in Table 3. 

### 3.5. Data Synthesis

#### 3.5.1. Precision and Accuracy of the Skeletal Method for BA Assessment among Children of Caucasian Ethnicities 

##### Precision of the Greulich and Pyle Atlas (GPA) Radiographic Skeletal Method

*Intra-examiner reliability*

The intra-examiner reliability of GPA for determining BA in Caucasian children was moderate to excellent, indicating high repeatability.

In Mediterranean countries, Pinchi et al. (2014) [50] in a retrospective observational study carried out in Florence (Italy) showed that the intra-examiner reliability of this method of determining CA was very high for both Caucasian boys (*r* = 0.907) (95% CI = 0.761–0.966, *p* < 0.05) and girls (*r* = 0.928) (95% CI = 0.789–0.977, *p* < 0.05) In the same country, Santoro et al. (2012) [63] in a study with southern children aged 7 to 15 yrs also found that the inter-examiner reliability of the GPA was moderate (*r* = 0.88) (*p* < 0.0001) for boys while for girls it was slightly lower (*r* = 0.81) (*p* < 0.0001). Additionally, in Portuguese children aged 12 to 10 yrs belonging to this ethnic group, Santos et al. (2011) [61] confirmed high intra-examiner reliability (*r* = 0.99) (*p* < 0.05) when using the GPA method to assess BA.

In other studies of Caucasians in Northern Europe, Kullman (1995) [69] found moderate intra-examiner reliability for GPA (*r* = 0.64–0.74) in determining the CA in a sample of Swedish children aged 12 to 19 yrs. On the other hand, Hackman and Black (2013) [57,62] found that in Scottish children and adolescents under 21 yrs of age, the intra-examiner reliability of the GPA to quantify age was excellent (*r* = 0.969) (*p* < 0.001).

In Lower Saxony (Germany), Schmidt et al. (2007) [67] showed that GPA was highly correlated as a method of identifying changes in CA (*r* = 0.96) (*p* < 0.05) for boys and (*r* = 0.96) (*p* < 0.05) for girls. In children aged 5 to 19 yrs from Rotterdam in the Netherlands, Van Rijn et al. (2001) [68] determined that the Pearson’s correlation coefficient was *r* = 0.979 for males (*p* < 0.001) and *r* = 0.974 (*p* < 0.001) for female girls, indicating high precision in estimating CA.

These results are similar to those of other studies carried out in other Anglo-Saxon countries. On the one hand, in a prospective cohort of Caucasian children in the United States, Calfee et al. (2010) [80] identified that the intra-examiner reliability of the GPA to estimate CA was moderate at *r* = 0.890 (*p* < 0.001). On the other hand, Maggio, Flavel, Hart, and Franklin (2016) [68] reported a very high repeatability and a very strong Pearson’s correlation coefficient between BA and CA in boys (*r* = 0.970) and in girls (*r* = 0.972) in Australia.

*Inter-examiner reliability or concordance*

Inter-examiner reliability of the GPA for assessing BA in Caucasian children ranged from low to high, suggesting controversy over its reproducibility. In France, the agreement of the GPA method measured by the intraclass correlation coefficient (*ICC*) was excellent for this ethnic group (*ICC* = 0.94) (95% CI: 0.91–0.96, *p* < 0.05) (64) For the United Kingdom, Alshamrani et al. (2020) [55] found small differences in concordance based on sex in a sample of British Caucasian children, showing that females had lower intraclass correlation coefficients (*ICC* = 0.984) than males (*ICC* = 0.991). In America, Calfee et al. (2010) found excellent concordance with GPA, *ICC* = 0.982, when estimating CA in a sample of Caucasian children in the northwestern United States [80].

On the other hand, the concordance of the GPA method using Cohen’s kappa coefficient estimation seems to be controversial. In Europe, this method has been used to determine CA in Caucasian Portuguese girls under 13 yrs of age. Martinho et al. (2021) [60] found that inter-observer reliability was low, at *k* = 0.48 (*p* < 0.05) Also, in France, Zabet et al. (2014) [65] found in a sample of Caucasian children from the city of Tours aged 10 to 19 yrs that Cohen’s kappa coefficient applied to the GPA showed an inter-examiner reliability of *k* = 0.96 (*p* = 0.0177).

In the Middle East, Soudack et al. (2012) [54] also reported lower agreement among examiners for GPA, at *k* = 0.371 (*p* = 0.0177), in a sample of Caucasian children at the Edmond and Lily Safra Children’s Hospital in Tel Aviv (Israel). Within the same sample, girls had significantly higher levels of agreement (*k* = 0.4667) (*p* = 0.005). Along the same lines, Büken et al. (2007) [40] published a lower inter-examiner reliability when using GPA to estimate CA in Turkish boys (*k* = 0.275) (*p* <0.001) and for girls (*k* = 0.143) (*p* <0.001) of Caucasian ethnicity. By contrast, Maggio, Flavel, Hart, and Franklin (2016) [76] published a study of Caucasian children in Perth, Australia, which showed that when determining CA using the GPA method, there was great agreement among examiners (*k* = 0.887, *p* < 0.001).

Finally, Alcina et al. (2017) [58] showed through Lin’s correlation coefficient of agreement (*ρc* = 0.99) that the repeatability of the GPA method in Hispanic Caucasian children aged 0 to 18 yrs is excellent regardless of the gender of the sample.

##### Accuracy of the Greulich and Pyle Atlas (GPA) Radiographic Skeletal Method

*Mean differences*

In terms of accuracy, that is, the capability of the BA estimation method to determine CA, the included studies generally support its use in Caucasians, although they found slight underestimation in children.

Mansourvar et al. (2013) [23], in a retrospective study with Caucasians living in Malaysia, found that the accuracy of the method was very good for children aged 10 to 16 yrs (MD = 0.044 yrs, *p* > 0.05). By contrast, although Kullman (1995) [69] confirmed its accuracy in Swedish children, he identified that GPA underestimated CA (MD = 0.4 yrs, *p* > 0.05). Similarly, among Caucasian children from Montpellier (France), GPA underestimated CA by a magnitude of MD = 1.27 mos (SD = 1.56, *p* < 0.05) [64]. Also in northern France, Zabet et al. (2014) showed that in Caucasian children, GPA slightly underestimated the CA (MD = 2.29 mos, SD = 10, *p* < 0.05).

A retrospective study by Santoro et al. (2012) conducted with a Caucasian population in southern Italy found that GPA slightly underestimated CA for both males (MD = 0.1 yrs, SD = 1.3, *p* = 0.18) and females (MD = 0.4 yrs, SD = 1.0, *p* < 0.0001) aged between 7 and 15 yrs [63]. Santos et al. (2011) [61] studied accuracy in Caucasian Portuguese children aged 12 to 20 yrs living in the city of Coimbra and found that the GPA underestimated the CA of the participants in a range from 2 to 7 mos (*p* < 0.05).

Similarly, Schmidt et al. (2007) [67] showed that this method of estimating CA underestimated the age of Caucasian boys (MD = 0.49 yrs, SD = 2.02, *p* < 0.05) and girls (MD = 0.39 yrs, SD = 2.16, *p* < 0.05) from northwestern Germany.

Wenzel et al. (1984) found statistically significant differences between CA and BA for GPA in Austrian boys from Graz (*p* < 0.01) but no differences in adolescent girls aged between 7 and 16 yrs (*p* = 0.4) were determined. Groell et al. (1999) [66] also found no statistically significant differences between bone and CA, although they discovered an underestimation of BA in the same ethnic group (MD = 0.4 mos, SD = 4.0 in boys; MD = 1.1 mos, SD = 5.9 in girls, *p* = 0.20).

Alshamrani et al. (2020) agreed with these results and found that GPA underestimated CA by 4 months (*p* < 0.01) in Caucasian males [55] from Sheffield (United Kingdom). When analyzed by age, the Scottish study by Hackman and Black (2013) published that in males aged 0–2 yrs, the GPA underestimates the CA from 0.2 to 10 mos (*p* < 0.05). A similar situation occurs in children aged 0 to 10 yrs for whom the method underestimated their age from 2.44 to 3.54 mos (*p* < 0.05).

Paradoxically, the same authors noted that the situation reverses between 11 and 15 yrs, during which time the GPA overestimates the CA by 1.74 mos (*p* < 0.05). The observed mean differences increased in the order of 1.62–11.05 mos in male adolescents aged 13 to 17 yrs (*p* < 0.05) while in girls aged 9–17 yrs, the overestimation reached an interval of 0.20 to 5.73 mos (*p* < 0.05) [57].

Also, in a Spanish study by Ebri (2021) [59], when comparing the accuracy of the GPA relative to the Ebri carpal index (EOIC) it was found that the GPA overestimated BA by almost 6 mos. In the same work, when comparing the accuracy of GPA relative to the carpo-metacarpal-phalangeal index (EOICMF) it was observed that the GPA overestimated BA by almost 6.5 mos. Comparing the accuracy of the GPA with EOIMF, it was observed that the GPA overestimated BA by almost 5 mos.

In the Middle East, Cantekin et al. (2012) [41] found among Caucasian children in eastern Turkey that the GPA method slightly underestimated the CA of participants (MD = 0.13 yrs, 95% CI: 0.31–0.70 yrs, *p* > 0.05). Based on children aged 10 to 17 yrs, there was a delay between the age scored by the GPA and the CA in Caucasian children from eastern Turkey, with a mean difference of 0.02 yrs for the youngest ages and 0.24 yrs for those on the verge of of adulthood. For Turkish girls of Caucasian ethnicity, these differences were higher within the same age group, reducing the mean difference between the GPA and CA by 0.03 yrs in children of 17 yrs.

In this sense, the GPA underestimated CA in Caucasian children aged 9 to 17 yrs in the Malatya and Sivas regions of the Anatolian peninsula and found a difference in the accuracy for males (MD = 1.19 mos, 95% CI: 12.81 ± 2.3 mos, 13.71 ± 2.6 mos, *p* < 0.05) compared to women (MD = 0.90 mos, 95% CI = 12.91 ± 2.3 mos, 14.11 ± 2.6 mos, *p* < 0.05) [43] In the same region, the GPA underestimated CA in Caucasian children from Tel Aviv (Israel) aged 15 to 18 yrs, finding a relevant difference for males (MD = 2.9 mos, 95% CI, *p* < 0.0043). [54,77]

If we analyze the results from Australia, the accuracy of the GPA was measured for Caucasian children of this country and a slight underestimation of BA was found for both boys (MD = 1.5 mos, *p* = 0.142) and girls (MD = 3.7 mos, *p* = 0.002). [77] Strikingly, if we analyzed by age, the GPA method underestimated (MD = 0.81 mos, *p* = 0.719) the BA during early childhood but as children grew, the GPA overestimated the BA (MD = 3.8 mos, *p* = 0.001).

##### Sensitivity and Specificity of the Greulich and Pyle Atlas (GPA) Radiographic Skeletal Method

Regarding the sensitivity of the GPA, a value of 90% was found for boys and 87.71% for women. Regarding the specificity of this radiological method for BA assessment, it was found to be 87.18% for boys and 82.76% for Caucasian girls from Italy. [62]

##### Precision of the Tanner–Whitehouse 2 and 3 (TW2 and TW3) Radiographic Skeletal Methods

*Intra-examiner reliability*

Among the included studies, the intra-examiner reliability of TW2 and TW3 in determining BA in Caucasian children was excellent, indicating high repeatability. Some retrospective studies, such as that by Pinchi et al. (2014) [62] found that the intra-examiner reliability of TW2 was very high for both boys (*r* = 0.862, 95% CI = 0.759–0.949, *p* < 0.05) and girls (*r* = 0.929, 95% CI = 0.793–0.978, *p* < 0.05)

For TW3, this same work conducted with Caucasian Italian children showed that the intra-examiner reliability of TW3 was also high for male children (*r* = 0.843, 95% CI = 0.617–0.942, *p* < 0.05) as for female children (*r* = 0.910, 95% CI = 0.817–0.956, *p* < 0.05).

##### Accuracy of the Tanner–Whitehouse 2 and 3 (TW2 and TW3) Radiographic Skeletal Methods

*Mean differences*

Ebri (2021) showed the accuracy of the TW2 in assessing the CA of Caucasian Hispanic children using the mean difference between TW2 and various anthropometric indices validated in this population. For the carpo-metacarpal-phalangeal index (EOICMF), TW2 was observed to overestimate CA by nearly 4 mos and 6 mos (*p* > 0.05) with little difference between sexes. Similar results were found when analyzing differences in CA using TW2, the metacarpal-phalangeal index (EOIMF), and the Ebrí-carpal index (EOIC) where the age of boys was found to be overestimated by 5 mos [59].

##### Sensitivity and Specificity of the Tanner–Whitehouse 2 and 3 (TW2 and TW3) Radiographic Skeletal Methods

Pinchi et al. (2014) published that the sensitivity of TW2 in Italian Caucasian children was 100% in males and 87.50% in females, whereas the specificity was 72.92% in boys and 72.41% in girls. The same study conducted by the University of Florence (Italy) found that the sensitivity of TW3 in white Italian children was 90% for boys and 71.42% percent for girls, while the specificity was 87.5% in boys and 83.87% in girls [62].

##### Precision of the Demirjian Radiographic Dental Method 

Dental methods for estimating CA are not precise and accurate enough to replace a skeletal radiographic method. However, despite this, AP radiographs of the carpus and left wrist can be a valid alternative for determining CA when they are uninterpretable.

*Intra-examiner reliability*

The work of Santoro et al. (2012) found in terms of the accuracy of the Demirjian method that the intra-examiner reliability calculated by Pearson’s correlation coefficient was *r* = 0.77, indicating that the precision of this dental method in detecting CA changes was moderate [63].

##### Accuracy of Kullman’s Radiographic Dental Method 

*Mean differences*

When analyzing the accuracy of Kullman’s (1995) [69] dental method for determining CA in a sample of Caucasian children, it was observed that the method detected statistically significant differences between them (MD = 1.2 yrs, SD = 1.0–1.4, *p* < 0.05).

#### 3.5.2. Precision and Accuracy of the Skeletal Method for BA Assessment among Children of Asian ethnicities 

##### Precision of the Greulich and Pyle Atlas (GPA) Radiographic Skeletal Method

*Intra-examiner reliability*

The GPA method for Asian children showed very high intra-examiner reliability, with a Pearson’s correlation coefficient of *r* = 0.94 (*p* < 0.001) for a sample of Korean children aged 7 and 12 yrs old [50].

*Inter-examiner reliability or concordance*

Regarding concordance, Chiang and Lin (2005) [52] found that the GPA had excellent inter-observer reliability *k* = 0.997 (*p* < 0.05) when used to calculate the CA of 10-year-old Taiwanese children.

##### Accuracy of the Greulich and Pyle Atlas (GPA) Radiographic Skeletal Method

*Mean differences*

Regarding accuracy, studies have maintained the applicability of the GPA in this ethnic group, although they found deviations towards overestimation of BA associated with CA in children.

The radiology the GPA method appears to be sufficiently accurate in estimating the CA of children. In a sample of X-rays of the carpal and left wrist bones from Chinese children aged 3 to 6 yrs in Zhejiang Province, Gao et al. (2022) [48] found that the GPA method was 12.02% accurate in determining CA for boys and 25.76% for girls.

Furthermore, Mansourvar et al. (2014) [23] found in a retrospective study that the GPA significantly overestimated CA in 4-year-old Malay children, with a mean difference between CA and BA of 2.3 mos (*p* < 0.05).

In South Korean children, in a sample of carpal and left wrist radiographs the GPA was shown to slightly overestimate the CA (MD = 0.45 mos, SD = 1.79) [50]. In this country, the GPA significantly overestimated CA in participants younger than 18 yrs of age. According to a report by Oh et al. (2012), the BA method overestimated CA by 54.6% in boys and 74.3% in girls [51]. Moreover, Chiang and Lin (2005) [52] showed that when applying the GPA to a cohort of Taiwanese girls aged 9 to 17 yrs, CA was overestimated by 0.18 to 1.48 mos (*p* < 0.05).

In contrast to these results, under the same study conditions as in Taiwanese children aged 13 to 18 yrs, the same authors found that the GPA underestimated CA by between 0.13 and 1.28 yrs of age (*p* < 0.05).

##### Precision of the Tanner–Whitehouse 3 (TW3) Radiographic Skeletal Method

*Intra-examiner reliability*

In terms of repeatability, TW3 showed very strong intra-examiner agreement, as evidenced by a Pearson’s correlation coefficient of *r* = 0.93 (*p* < 0.001) for a sample of Korean boys and girls aged 7 and 12 yrs old [50].

##### Accuracy of the Tanner–Whitehouse 3 (TW3) Radiographic Skeletal Method

*Mean differences*

On the other hand, Gao et al. (2022) [48] found that in samples of radiographs from Chinese children aged 3 to 6 yrs old the accuracy of the TW3 method was lower, 32.24% for boys and 24.15% for girls.

Among South Korean boys and girls, TW3 overestimated CA in participants under 18 yrs by 59.6% in boys and 72.2% in girls [51]. In the same region, TW3 slightly overestimated CA in Korean children (MD = 0.45 mos, SD = 1.81 mos) [50].

Griffith, Cheng, and Wong (2007) [49] observed in a sample of Chinese children that when analyzing carpal and left wrist radiographs of children aged 6 to 18 yrs with TW3, it was found that the TW3 statistically significantly overestimated CA compared to the GPA method (*p* < 0.0001).

##### Precision of the Korean Standard Chart (KS) Radiographic Skeletal Method

*Intra-examiner reliability*

The Korean Standard BA Chart (KS) method studied by Griffith, Cheng, and Wong (2007) [49] showed high intra-examiner reliability with a Pearson’s correlation coefficient of *r* = 0.94 (*p* < 0.001) for a sample of Korean children aged 7 to 12 yrs.

##### Accuracy of the Korean Standard Chart (KS) Radiographic Skeletal Method

*Mean differences*

In terms of accuracy, Kim, Lee, and Yu (2015) found that the KS slightly overestimated CA (MD = 0.21 mos) (SD = 1.19 mos, *p* < 0.05) in a sample of AP radiographs of the carpi and left wrists of Korean children [50].

##### Accuracy of the RUS-CHN (China 05) Radiographic Skeletal Methods

*Mean differences*

Other specific methods for determining CA in Asian child populations, such as RUS-CHN (China 05), had lower accuracy, 12.02% for boys and 21.26% for girls [48].

#### 3.5.3. Precision and Accuracy of the Skeletal Method for BA Assessment among Children of Indian ethnicities 

##### Precision of the Greulich and Pyle Atlas (GPA) Radiographic Skeletal Method

*Intra-examiner reliability*

The precision of the GPA is high, as shown in the study by Patel et al. (2015) [36]. It has been identified for the case of Indian children aged 6 to 16 yrs living in the region of Gandhinagar (India) that the precision between CA and BA was 90.65%, while correlation with BA was also very strong (r = 0.921, *p* < 0.001).

*Inter-examiner reliability or concordance*

The inter-examiner reliability of the GPA method was very high (*k* = 0.82) (*p* < 0.01) when looking at the scores obtained by different observers in determining CA from a sample of wrist and left hand radiographs of 10-year-old Thai children [79].

Analyzing the reproducibility of the GPA among Hindu children from Mumbai, Keny et al. (2017) [35] showed that this method had good inter-examiner agreement between raters with a *k* = 0.68 (95% CI = 0.504–0.848, (*p* < 0.001), albeit substantially lower than for other Indo-European ethnicities.

GPA agreement was determined based on the intra-class correlation coefficient for Malay boys and girls aged between 9 and 18 yrs. The concordance among observers was excellent in determining CA for boys with *ICC* = 0.947 (*p* = 0.86) and females with slightly lower *ICC*= 0.93 (*p* = 0.33) [78].

##### Accuracy of the Greulich and Pyle Atlas (GPA) radiographic skeletal method

*Mean differences*

In terms of accuracy, the studies included in this review stated that the skeletal methods GPA and TW3 slightly overestimated BA when compared with CA in Indian children.

In a sample of Indian children between 1 and 15 yrs old, Keny et al. (2017) [35] found that GPA overestimated CA at MD = 10 mos for males aged 1 to 6 yrs. Along the same lines, the differences appeared to be slightly smaller in girls, with differences between CA and BA lasting up to 8 mos.

Similarly, in their work, Patil et al. (2012) [37] concluded that the GPA overestimated CA in Indian children. For boys aged 8 to 9 yrs, there was a significant difference between CA and BA (MD = 2.11 yrs, *p* < 0.05), whereas this difference seemed to decrease with bone maturity (MD = 1.33 yrs, *p* < 0.05). Similar results were found in young girls aged 4 to 8 yrs, with a mean difference of CA MD = 0.52 yrs (*p* < 0.05) and MD = 0.22 yrs (*p* < 0.05) for 18-year-olds.

Among Hindu children aged 1 to 19 yrs in Eastern Uttar Pradesh, the GPA slightly overestimated CA (MD = 0.56 mos, SD = 1.33 yrs, *p* = 0.001). These differences are not uniform, as males had MD = 9.03 mos (SE = 0.25, t = 2.98, *p* ≤ 0.05) which was significantly smaller in females (MD = 4.33 mos, SE = 0.18, *p* ≤ 0.05). [39] As the children grew, Tiwari et al. (2020) showed a slight decrease in the mean differences of 0.89 yrs (SD = 0.85 yrs, *p* = 0.03) for 0 to 5 yrs, and 0.81 yrs (SD = 1.57 yrs, *p* = 0.03) for 0 to 15 yrs.

The accuracy of the GPA in determining BA in Malay children was weaker relative to CA, as at least one mean difference has been detected due to underestimation (MD = 0.6 yrs, 95% CI, *p* < 0.05) in males and (MD = 0.7 yrs, 95% CI, *p* < 0.05) in females. If we analyzed by age, we found that CA is overestimated in adult children. Therefore, the difference between CA and BA increased starting from MD = 0.6 yrs (*p* < 0.05) for the ages of 13 to 13.9 yrs to MD = 1.5 yrs (*p* < 0.05) in children from 18 to 18.9 yrs [78].

##### Precision of the Tanner–Whitehouse 3 (TW3) Radiographic Skeletal Method

*Inter-examiner reliability or concordance*

The inter-observer concordance reliability of the TW3 RUS method was very high, with *k* = 0.66–0.88 (*p* < 0.01), in radiographs of the wrist and left hand in 10-year-old Thai children [79].

##### Precision of the Fishman Radiographic Skeletal Method

*Intra-examiner and Inter-examiner reliability or concordance*

The degree of intra-examiner agreement for the Fishman method for the skeletal determination of CA was very good (k = 0.91) (*p* < 0.01). On the other hand, the inter-observer reliability of the Fishman method for CA was very good with *k* = 0.85 (*p* < 0.01) in 18-year-old Thai children [79].

##### Accuracy of McKay’s (MK) Radiographic Skeletal Method

*Mean differences*

Keny et al. (2017) [35] examined the CA accuracy of McKay’s skeletal radiological method and showed that in Mumbai Indian children aged 1 to 6 yrs it overestimates age by 22 mos for boys and 17 mos for girls.

##### Precision of Demirjian’s Radiographic Dental Method 

*Intra-examiner reliability*

Demirjian’s dental method showed high accuracy with a moderate linear correlation coefficient between CA and BA of *r* = 0.882 (*p* < 0.001) in boys and very strong *r* = 0.956 (*p* < 0.001) for girls evaluated with this method [36].

##### Accuracy of Demirjian’s Radiographic Dental Method 

*Mean differences*

The accuracy of Demirjian’s dental method was confirmed by Patel et al. (2015) [36] who found that CA was overestimated in Indian children aged 6 and 10.99 yrs (*p* > 0.05) while being underestimated in adolescents aged 11 to 14.99 yrs (*p* > 0.05).

##### Precision of Willem’s Radiographic Dental Method

*Intra-examiner reliability*

Patel et al. (2015) [36] studied another dental method, namely Willem’s, that presented a high precision in detecting changes in CA from the age of tooth wear morphology with a correlation coefficient of *r* = 0.959 (*p* > 0.05).

##### Precision of other Radiographic Methods

Cervical vertebrae maturation (CVM)

*Intra-examiner reliability*

In a sample of cervical radiographs from Indian children aged 8 to 14 yrs, Prasad et al. (2013) [38] observed that the cervical ripening method was highly accurate in detecting possible changes in CA, with a linear correlation coefficient of *r* = 0.915 (*p* = 0.000).

##### Accuracy of Other Radiographic Methods

*Mean differences*

The mean difference between the estimated CAs based on cervical ripening (CMV) studied by Prasad et al. (2013) [38] slightly overestimated the CA of Indian children (MD = 0.097 yrs) (SD = 0.793 yrs, *p* > 0.05). The same author found a slightly larger difference when estimating CA (MD = 0.170 yrs, SD = 1.08 yrs, *p* > 0.05) from BA for children of this ethnic group using TW3.

#### 3.5.4. Precision and Accuracy of Skeletal Methods for BA Assessment among Children of Arab ethnicities 

##### Precision of the Greulich and Pyle Atlas (GPA) Radiographic Skeletal Method

*Intra-examiner reliability*

The intra-rater reliability of the GPA in determining the CA of Arab children ranged from moderate to high. X-rays of Pakistani children from Karachi were strong with a positive linear association of *r* = 0.915 (*p* < 0.001) and *r* = 0.943 (*p* < 0.001) for boys and girls, respectively [44].

On the other hand, in Arab children from Saudi Arabia, the repeatability of the GPA was demonstrated by the identification of a strong association between CA and BA with *r* = 0.873 (*p* < 0.001) and *r* = 0.872 (*p* < 0.001) for boys and girls, respectively [46].

In addition, in a sample of young Pakistanis aged 0 and 18 yrs, the GPA showed excellent accuracy in quantifying the association between CA and BA obtained by this method (*r* = 0.992) (*p* < 0.001) [45].

However, the correlation between CA and BA estimated by the GPA determination method in native Pakistani children showed a positive and moderate association for both boys and girls (*r* = 0.778) (*p* < 0.001) [30,46].

*Inter-examiner reliability or concordance*

The intra-examiner agreement was excellent, reaching *ICC* = 0.995 in boys and *ICC* = 0.996 in girls [46]. The intra-examiner agreement was *ICC* = 0.991 for boys and *ICC* = 0.984 for girls [47]. In a sample of Pakistani children, excellent intra-examiner concordance was detected, reaching *ICC* = 0.998. [44]

An Israeli study indicated that in a sample of male children from Tel Aviv, as a method of determining BA, the GPA demonstrated an excellent degree of intra-examiner agreement (*ICC* = 0.9846). These results were similar in women with an excellent degree of intra-examiner agreement (*ICC* = 0.9787) [54].

##### Accuracy of the Greulich and Pyle Atlas (GPA) Radiographic Skeletal Method

*Mean differences*

The accuracy of the estimates of the GPA skeletal radiographic method among children of Arab ethnicity is controversial as there is no consensus on the results obtained in the studies included in this review.

On the one hand, for boys aged 4 to 8 yrs in Saudi Arabia, the CA tended to be significantly overestimated (MD = 143.5 mos, SD = 44.0, *p* < 0.001), while it was slightly less so for girls (MD = 116.9 mos, SD = 41.8, *p* < 0.001) [46]. Along the same lines, Moradi et al. (2012) [53], in a cross-sectional study, found that, in a sample of Iranian boys aged 6 to 18 yrs, the GPA overestimated CA more markedly in males (MD = 0.37, SD = 0.98 yrs, *p* > 0.05) than in girls (MD = 0.04) (SD = 0.78 yrs, *p* > 0.05).

Likewise, the accuracy of the GPA in quantifying CA in Pakistani children living in Karachi was good although it was slightly overestimated (MD = 0.4 mos, *p* = 0.584) when studied in young populations up to 18 yrs of age [45].

On the other hand, and contrary to the above, in a similar sample, but with Saudi children aged 10.48 ± 4.8 yrs, Alshamrani et al. (2020) [47] found that the GPA underestimated the CA of participants by 4 mos (*p* < 0.01).

##### Precision of the Tanner–Whitehouse 3 (TW3) Radiographic Skeletal Method

*Inter-examiner reliability or concordance*

The agreement between examiners of the TW3 method in Saudi children was very good, although it differed between sexes, as indicated by a lower intraclass correlation coefficient for men (*ICC* = 0.963) compared to women (*ICC* = 0.972) [47].

##### Accuracy of the Tanner–Whitehouse 3 (TW3) Radiographic Skeletal Method

*Mean differences*

In terms of accuracy, the TW3 for samples of children from Saudi Arabia with a mean of 10.21 to 10.48 yrs found an underestimation of CA of 2.5 mos (*p* < 0.01) [47].

##### Precision of Other Radiographic Methods

The Girdany and Golden’s method

*Intra-examiner reliability*

If we analyze the precision of Girdany and Golden’s method, there is a slight difference between sexes in CA determination, finding *r* = 0.865 for boys, and a greater correlation between BA and CA for girls, *r* = 0.909 [44].

*Inter-examiner reliability or concordance*

In Saudi children, the Girdany and Golden’s method showed a very high agreement between raters of *ICC* = 0.974 [44].

Cervical vertebrae maturation (CVM)

*Inter-examiner reliability or concordance*

In a sample of 14-year-old Turkish children, the inter-examiner agreement of the BA estimation method based on cervical maturity (CVM) was very good at *k* = 0.862–0.958 (*p* < 0.05). Other methods that studied inter-examiner agreement based on the stages of bone maturation of the hand and wrist (HWM) of Turkish children found that the degree of agreement between the different evaluators of the method was moderate (*k* = 0.812–0.961) (*p* < 0.05) [42].

##### Precision of Demirjian’s Radiographic Dental Method

*Inter-examiner reliability or concordance*

On the other hand, in this same sample of Turkish children, the Demirjian dental method of determining BA obtained a moderate degree of inter-examiner agreement, *k* = 0.823–0.928 (*p* < 0.05) [42].

#### 3.5.5. Precision and Accuracy of Skeletal Methods for BA Assessment among Children of Hispanic ethnicities 

##### Precision of the Greulich and Pyle Atlas (GPA) Radiographic Skeletal Method

*Intra-examiner reliability*

Regarding the accuracy of the GPA method, a strong and positive linear correlation was found (*r* = 0.890) (*p* < 0.001) that associated the increase in CA with an increase in BA scores obtained by this method in Hispanic children residing in the United States. [80] As such, the accuracy of the GPA determination method was very good, with an association coefficient between CA and GPA score of *r* = 0.918 (*p* < 0.05) found in a sample of antero-posterior radiographs of the left hand and wrist of Venezuelan children aged 6 to 12 yrs [81].

In Chilean children under 16 yrs of age, for the manual GPA score against an automated expert system, Pose et al. (2018) [83] found a very strong and positive linear correlation ranging from *r* = 0.91 to 0.93 (*p* < 0.05) which would indicate the reliability of the procedure even when using machine learning.

*Inter-examiner reliability or concordance*

In an American study conducted by Calfee et al. (2010) [80] with a sample of Hispanic children aged 12 to 18 yrs, an excellent inter-examiner reliability of *ICC* = 0.982 was found for the GPA method.

##### Accuracy of the Greulich and Pyle Atlas (GPA) Radiographic Skeletal Method

*Mean differences*

The accuracy of the GPA from a sample of left hand and wrist radiographs from Hispanic children is consistent across the various studies included in this review.

In Hispanic children aged 9.96 to 11.12 yrs, Pose et al. (2018) [83] found that the GPA underestimated CA (MD = 0.19 yrs, 95% CI: 0.13–0.25, *p* < 0.05) in a large sample of children treated in an orthopedic clinic in Santiago de Chile. Mansourvar et al. (2014), who applied this method in a sample of Hispanic children aged 15 to 18 yrs living in California (USA), also detected an underestimation of CA (MD = 0.094 yrs, 95% CI, *p* > 0.05) [23].

##### Precision of the Tanner–Whitehouse 3 Radiographic Skeletal Method

*Intra-examiner reliability*

Regarding the TW3 RUS method, López et al. (2008) [82] found in a sample of Venezuelan children a high accuracy in children aged 7 to 14 yrs (*r* = 0.91) (*p* < 0.05). Similar results were also obtained for girls in this sample with a Pearson correlation coefficient of r = 0.93 (*p* < 0.05). As for TW3 Carpal, which evaluates the regions of interest of the carpal bones, a lower accuracy was found, with *r* = 0.89 (*p* < 0.05) in boys and *r* = 0.82 (*p* < 0.05) for radiographs of girls.

##### Precision of Demirjian’s Radiographic Dental Method

*Intra-examiner reliability*

The accuracy of the Demirjian dental method found a very strong correlation coefficient *r* = 0.929 (*p* < 0.05) that associated CA with dental age obtained with the Demirjian method in a sample of Venezuelan children from Maracaibo in the State of Zulia between 6 and 12 years old [81].

#### 3.5.6. Precision and Accuracy of Skeletal Methods for BA Assessment among Children of African ethnicities 

##### Precision of the Greulich and Pyle Atlas (GPA) Radiographic Skeletal Method

*Intra-examiner reliability*

When the intra-examiner agreement was studied, a very strong correlation was observed between CA and GPA scores in boys (*r* = 0.93) (*p* > 0.05) and girls (*r* = 0.94) (*p* > 0.05) from the central district of Botswana [74].

Regarding the degree of intra-observer agreement, the GPA showed very strong correlations in radiographic samples of boys (*r* = 0.96) (*p* < 0.05) and for Zimbabwean girls (*r* = 0.96) (*p* < 0.05) [73].

In South Africa, in the prospective cohort study involving young Bantu people, Dembetembe et al. (2012) [71] found a moderate correlation when analyzing the accuracy of the GPA method of *r* = 0.76. However, when subjects were aged between 13 and 18.5 yrs, intra-examiner reliability dropped to a linear correlation of zero *r* = 0.02.

*Inter-examiner reliability or concordance*

When studying the inter-examiner concordance of the GPA method in South African children, an intraclass coefficient *ICC* = 0.99 was found, with a statistical significance of *p* < 0.001 [72]. In Botswana, intra-observer agreement of the GPA with left hand–wrist radiographs of male children between 5 and 18 yrs also registered an excellent *ICC* = 0.97 (*p* > 0.05), being somewhat higher for girls (*ICC* = 0.98) (*p* > 0.05) [74].

##### Accuracy of Radiographic Skeletal Method Greulich and Pyle Atlas (GPA)

*Mean differences*

The accuracy of GPA and TW3 among children of African ethnicity is controversial because these radiological methods overestimated CA in children of African descent.

In South African children, the GPA was less accurate in determining BA relative to CA, with at least one mean difference overestimation of MD = 7.4 mos (SD = 15.7 mos, *p* < 0.05) found [72].

Among African males younger than 19 yrs, the mean difference was even more overestimated than reported in previous studies, with a CA of MD = 4.4 ± 14.5 mos (95% CI, *p* < 0.05). For African women under 18 yrs, the mean differences were MD = 2.4 ± 12.8 mos (95% CI, *p* < 0.05) [72].

Another study found that the GPA method overestimated age in African adolescent males from Zimbabwe and found important differences between CA and BA (MD = 0.76 yrs, 95% CI: −0.95, −0.57, *p* < 0.05) [73]. Similar results were found in a study conducted in Botswana, in which the differences between CA and BA after using the GPA were accentuated as the CA increased from (MD = 0.25 yrs, *p* < 0.05) from 5 to 10 yrs to (MD = 0.94 yrs, 95% CI, *p* < 0.05) in children aged between 15 and 18 yrs [74].

In Ethiopia, the GPA was also found to increase CA in males (MD = 8.7 mos (*p* < 0.05) and MD = 11.8 mos (*p* < 0.05) and females between 10 to 22 yrs [75]. Even in the African American population, the GPA method overestimated CA at 15 yrs of age (MD = 2.4 yrs, 95% CI, *p* > 0.05) [23].

##### Precision of the Tanner–Whitehouse 3 Radiographic Skeletal Method

*Intra-examiner reliability*

Intra-examiner reliability of the TW3 RUS was similar to that of the GPA and showed strong to very strong correlations for boys (*r* = 0.95) (*p* < 0.05) and for Zimbabwean girls (*r* = 0.93) (*p* < 0.05) [73].

##### Accuracy of the Tanner–Whitehouse 3 Radiographic Skeletal Method

*Mean differences*

When studying the accuracy of this method for age determination in Zimbabwean children, the mean was found to be overestimated (MD = −0.43 yrs, 95% CI: −0.61, −0.24, *p* < 0.05) [73].

## 4. Discussion

The purpose was to identify, evaluate, and summarize the results of relevant individual studies regarding the precision and accuracy of radiographic assessment procedures for BA in children of different ethnicities. Considering the results obtained, it can be stated that radiological methods such as the GPA or TW3 are generally considered precise for all ethnic groups, as evidenced by the intra-examiner reliability results and the excellent inter-examiner agreement [85].

However, the existence of various results regarding accuracy raises several technical, legal, and ethical issues for pediatric and forensic practice in determining BA. In this sense, we cannot ignore that currently the determination of BA must be based on the assessment of skeletal developmental milestones using radiography, and therefore, given the apparent racial bias, the resulting decisions may be inappropriate [86].

### 4.1. Skeletal Methods for BA Assessment among Children of Caucasian ethnicities 

GPA precision showed high concordance among British Caucasian children [55] and southwestern Australians of British ancestry [76]. When the agreement among observers is measured only with Cohen’s kappa coefficient it is observed that for Caucasians from Israel [54] and Portugal [60] inter-examiner reliability was weaker than for Anglo-Saxons. This can be explained by the fact that the Caucasian American population of Northern European origin used by Greulich and Pyle to develop their atlas was different from Mediterranean Caucasians.

Analyzed by gender, there are differences in intraclass correlation coefficients when using the GPA. Based on the study of Alshamrani et al. (2020b) [55], this coefficient is higher for boys than for girls, which in principle would suggest that the GPA is more accurate in boys than in girls. These results are also consistent with the findings of Nang et al. (2023) [78] for Indian children; however, they contrast with those obtained by several authors for the Arab [44,46,47] and African [74] populations in which inter-examiner agreement was stronger in girls than boys. This could be due, among other causes, to differences between the study samples, as we found, contrary to Alshamrani et al. (2020b) [55], whose girls had an average age of 8.8 yrs (SD = 3.6), because they included older girls, resulting in greater precision by showing more ossification regions.

Regarding accuracy, we can point out that applying the GPA to Caucasian children generally leads to underestimation of CA. We found that the underestimation is lower in the study by Mansouvar et al. (2014) [23] conducted on a child population from the Children’s Hospital Los Angeles (United States), which is to be expected considering that the reference population of the GPA is also American [57]. Likewise, the underestimation of the GPA remains lower for Scottish children, although in this case the interval of mean difference is greater. This could be explained by the existence of common ancestors between the Scottish population and the northern American population from which the Greulich and Pyle method originates.

By contrast, the underestimation of CA is much more pronounced among Caucasian children from Central European [66,67,70] and Scandinavian countries [69]. If we analyze by gender, we find a lower underestimation in Turkish [43] compared to German girls [67,70], in which the greatest deviation is observed. Among boys, Turkish children have the lowest underestimation [43], while Austrians have the greatest downward deviation [59] On the contrary, the GPA radiological method, and also Tanner–Whitehouse 2 and 3, resulted in an overestimation of CA for Spanish children’s CA [59] as well as Scottish children between 9 and 17 yrs, possibly due to a peculiar growth pattern of this populations [57].

### 4.2. Skeletal Methods for BA Assessment among Children of Asian ethnicities 

Although the GPA precision was high for the Asian population as measured by intra- and inter-examiner reliability, we found that accuracy for CA was lower for this ethnic group. The highest overestimation was found in Malaysian children [23] and the lowest in South Korea [50]. By age, the overestimation of the Taiwanese population [52], was highest among children aged 9 and 17 yrs, and slightly lower among children aged 13 and 18 yrs. In the gender analysis, the GPA tends to overestimate more for Korean girls [51] and less for Chinese boys [48].

### 4.3. Skeletal Methods for BA Assessment among Children of Indian Ethnicities 

In Indians, GPA application leads to an overestimation of CA as observed with the accuracy of Demirjian’s dental method and cervical vertebrae maturation (CVM). This overestimation was evident among Hindus in the study by Patil et al. (2012) [37] while in children from the eastern Uttar Pradesh region, the method had one of the lowest overestimations [39]. We also note that this radiological BA assessment method leads to overestimation because the sample size depends on the age period we are referring to, with the mean difference being greater in children aged 0 to 5 in comparison to children aged 0 to 15 years [39] and even younger [37]. Overestimation of CA is generally higher in boys than in girls, with sexual difference being particularly pronounced among children in eastern Uttar Pradesh [39]. It stands out that this overestimation is minimized only in the group of girls aged 18 yrs or older [37]. For Malaysian ethnicity, the GPA overestimates the CA of those who have reached adult age and overestimates the CA of children aged 13 to 13.9 yrs [78]. In this group, unlike Hindu children, there is an underestimation of CA for both sexes, which is significantly higher for girls [78].

### 4.4. Skeletal Method for BA Assessment among Children of Arab Ethnicities 

In the Pakistani population, there is a discrepancy regarding the precision of the GPA, given the differences found in intra-examiner reliability [30,46]. It was also observed that when the same parameters were analyzed but using the Girdany and Golden’s method, a stronger correlation was found in girls than boys [44]. Furthermore, in terms of accuracy, the GPA overestimated the CA of children from Saudi Arabia [46], however, Alshamrani et al. (2020) found that GPA underestimated CA, as did the application of TW3 [47]. These results are consistent with those from a sample of wrist and left hand radiographs from Pakistan in which overestimation was minimal [45]. For Iranian Arab children, this overestimation was significantly smaller than the results reported by Albaker et al. (2021) for Saudi children, in which case there is a clear difference in CA overestimation in boys, which was 10 times higher than in girls [53].

### 4.5. Skeletal Methods for BA Assessment among Children of Hispanic Ethnicities 

The accuracy of GPA in radiographs of the left hand and wrist radiographs of Hispanic children was consistent across studies included in this review. As age increases, the underestimation of the GPA method decreases [23,83]. If we analyze by sex, applying TW3 RUS on radiographs of girls was more accurate than applying TW3 carpal.

### 4.6. Skeletal Method for BA Assessment among Children of African Ethnicities 

Furthermore, almost half of the included studies (*n* = 23, 45.90%) have been carried out with samples of Caucasian children while on the opposite side, we find the studies carried out in African children (*n* = 8, 4.18%). In general, the accuracy when applying the GPA and TW3 is low. This is attributed to the overestimation that occurs when applied to the African population. African youth living in the United States showed the greatest overestimation [23] compared with residents of Botswana [74] although the former had a smaller sample of X-rays, making comparability difficult.

As age increases, mean differences associated with CA appear to increase, with higher overestimation in adolescents than in children [23,72,73,74,75]. If we perform the analysis by sex, this previously described phenomenon remains, since the overestimation of CA increases if the samples include order boys and girls [72,75]. Specifically, we observed that by ethnic group, children of African ancestry yield the highest overestimation when using radiological BA assessment procedures. We also noted a bias in the mean age of this population, which may be due to the extremely small number of radiographs that comprised the sample and the wide age range of African children included in these studies [72].

### 4.7. Limitations

Our study has some methodological limitations that may affect the external validity of the results presented. Regarding the study design of the included studies, we must note that there was a high proportion of cross-sectional studies *n* = 19 (37.25%) which may become a limitation by preventing the study of the temporal sequence or the spatial references of ossification during the bone maturational process. As a consequence of these designs, the results obtained by the metric properties of radiological BA assessment methods can vary significantly.

In relation to the sampling of the studies, we observed some relevant aspects. First, the sampling techniques chosen by the authors were non-probabilistic and non-consecutive sampling. Second, within some ethnic groups, there was an imbalanced sample size in the number of male children recruited compared with girls, which may indicate a gender bias that alters the interpretation of the metric properties of the radiological methods. [86]

Moreover, almost half of the included studies (*n* = 23, 45.90%, *n* = 9777 AP left hand-wrist radiographs) were conducted on Caucasian children, whereas on the other hand, we found few studies carried out in African children (*n* = 8, 4.18%, *n* = 810 AO left wrist–carpal radiographs). This imbalance may lead to interpretation biases because there is no homogeneous group to compare the precision or accuracy of every radiological method [87].

Additionally, poor sample robustness is particularly important when the authors analyze the consistency of their method in multiethnic samples such as those including African Americans or Hispanic Americans. On this subject, we argue that estimates of BA in these groups without a dominant ethnic group are necessarily inaccurate [88,89].

Another limitation of these studies is that they do not allow us to know whether the observed differences between BA measurements and CA in each ethnic group are truly significant from a clinical perspective. This makes it less interesting for recipients of radiological age determination instruments, who are essentially legal medicine and pediatric professionals.

With this design, the level of organization to which these accuracy errors can be attributed is not clear to us, that is, whether they are the product of the individual, the family, or the ethnic group. It is evident that the results of bone maturation, and consequently the ossification, are an extremely complex and yet unknown phenomenon involving environmental, hormonal, and genetic factors whose interaction may explain the differences in accuracy found in radiological BA determination tools.

## 5. Conclusions

Radiological skeletal BA assessment procedures GPA and TW3 are both precise among children of different ethnic groups, but their accuracy in estimating CA can be altered by racial bias. Furthermore, radiological dental and cervical bone age assessment methods are equally precise but less accurate than radiological skeletal bone methods.

## Figures and Tables

**Table 1:** Search strategy.

Search Data	Database	Search Equation
10 January 2023	MEDLINE (PubMed)	“Reproducibility of results” [Mesh] OR “Dimensional Measurements Accuracy” [Mesh] OR “Diagnostic Techniques and Procedures” [Mesh] OR “Diagnostic imaging” [Mesh] OR “Radiography” [Mesh] OR “Age Determination by Skeleton” [Mesh] OR “Bone matrix” [Mesh] OR “Carpal bones” [Mesh] OR “Radius” [Mesh] OR “Wrist” [Mesh] OR “Racial Groups” [Mesh] OR “Race factors” [Mesh] OR “White people” [Mesh] OR “Black people” [Mesh] OR “Hispanic or Latino” [Mesh] OR “Asian people” [Mesh] OR “Native Hawaiian or Other Pacific Islander”[Mesh] OR “American Indian or Alaska Native”[Mesh] OR “Pacific Island People”[Mesh] OR “Asian American Native Hawaiian and Pacific Islander”[Mesh] OR “Bone Maturity” [tw] “Skeletal Maturation” [tw] OR “Skeletal Age” [tw] OR “Age Measurement” [tw] OR radiograp * [tw] OR radiol * [tw]
10 January 2023	MEDLINE (PubMed)	“Reproducibility of results” [Mesh] OR “Dimensional Measurements Accuracy” [Mesh] OR “Diagnostic Techniques and Procedures” [Mesh] OR “Diagnostic imaging” [Mesh] OR “Radiography” [Mesh] OR “Radiography, panoramic” [Mesh] OR “Age Determination by Teeth” [Mesh] OR “Dentition” [Mesh] OR “Teeth” [Mesh] OR “Tooth” [Mesh] OR “Molar, Third” [Mesh] OR “Incisor” [Mesh] OR “Racial Groups” [Mesh] OR “Race factors” [Mesh] OR “White people” [Mesh] OR “Black people” [Mesh] OR “Hispanic or Latino” [Mesh] OR “Asian people” [Mesh] “Native Hawaiian or Other Pacific Islander”[Mesh] OR “American Indian or Alaska Native”[Mesh] OR “Pacific Island People”[Mesh] OR “Asian American Native Hawaiian and Pacific Islander”[Mesh] OR “bone age measurement” [tw] OR “Orthopantomography” [tw] OR “Bone Maturity” [tw] “Skeletal Maturation” [tw] OR “Skeletal Age” [tw] OR “Age Measurement” [tw] OR radiograp * [tw] OR radiol * [tw]
12 January 2023	Cochrane Library	([mh “Reproducibility of results” ] OR [mh “Dimensional Measurements Accuracy] OR [mh “Diagnostic Techniques and Procedures”] OR [mh “Diagnostic imaging”] OR [mh “Radiography”] OR [mh “Age Determination by Skeleton”] OR [mh “Bone matrix”] OR [mh “Carpal bone”] OR [mh “Radius”] OR [mh “Wrist”] OR [mh “Racial Groups”] OR [mh “Race factors”] OR [mh “White people”] OR [mh “Black people”] OR [mh “Hispanic or Latino”] OR [mh “Asian people”] OR [mh “Native Hawaiian or Other Pacific Islander”] OR [mh “American Indian or Alaska Native”] OR [mh “Pacific Island People”] OR [mh “Native Hawaiian or Other Pacific Islander”] OR Bone Matur*:ti,ab,kw OR Skeletal Age:ti, ab, kw OR Age Measurement:ti, ab, kw)
12 January 2023	Cochrane Library	([mh “Reproducibility of results”] OR [mh “Dimensional Measurements Accuracy] OR [mh “Diagnostic Techniques and Procedures”] OR [mh “Diagnostic imaging”] OR [mh “Radiography, panoramic”] OR [mh “Age Determination by Skeleton”] OR [mh “Dentition”] OR [mh “Teeth”] OR [mh “Tooth”] OR [mh “Molar, third”] OR [mh “Incisor”] OR [mh “Racial Groups”] OR [mh “Race factors”] OR [mh “White people”] OR [mh “Black people”] OR [mh “Hispanic or Latino”] OR [mh “Asian people”] OR [mh “Native Hawaiian or Other Pacific Islander”] OR [mh “American Indian or Alaska Native”] OR [mh “Pacific Island People”] OR [mh “Native Hawaiian or Other Pacific Islander”] OR Orthopantomography:ti,ab,kw OR Bone Matur *:ti,ab,kw OR Skeletal Age:ti, ab, kw OR Age Measurement:ti, ab, kw)
14 January 2023	CINAHL	(MH “Reproducibility of results” OR MH “Dimensional Measurements Accuracy OR MH “Diagnostic Techniques and Procedures” OR MH “Diagnostic imaging” OR MH “Radiography” OR MH “Age Determination by Skeleton” OR MH “Bone matrix” OR MH “Carpal bones” OR MH “Radius” OR MH “Wrist” OR MH “Racial Groups” OR MH “Race factors” OR MH “White people” OR MH “Black people” OR MH “Hispanic or Latino” OR MH “Asian people” OR MH “Native Hawaiian or Other Pacific Islander” OR MH “American Indian or Alaska Native” OR MH “Pacific Island People” OR MH “Asian American Native Hawaiian and Pacific Islander” OR bone matur * OR Skeletal Matur * OR Skeletal Age OR Age Measurement)
14 January 2023	CINAHL	(MH “Reproducibility of results” OR MH “Dimensional Measurements Accuracy OR MH “Diagnostic Techniques and Procedures” OR MH “Diagnostic imaging” OR MH “Radiography, panoramic” OR MH “Age Determination by Skeleton” OR MH “Dentition” OR MH “Teeth” OR MH “Tooth” OR MH “Molar, Third” OR MH “Incisor” OR MH “Racial Groups” OR MH “Race factors” OR MH “White people” OR MH “Black people” OR MH “Hispanic or Latino” OR MH “Asian people” OR MH “Native Hawaiian or Other Pacific Islander” OR MH “American Indian or Alaska Native” OR MH “Pacific Island People” OR MH “Asian American Native Hawaiian and Pacific Islander” OR “Orthopantomography” OR bone matur* OR Skeletal Matur * OR Skeletal Age OR Age Measurement)
20 January 2023	Web of Science (WOS)	“Reproducibility of results” [Mesh] OR “Dimensional Measurements Accuracy” [Mesh] OR “Diagnostic Techniques and Procedures” [Mesh] OR “Diagnostic imaging” [Mesh] OR “Radiography” [Mesh] OR “Age Determination by Skeleton” [Mesh] OR “Bone matrix” [Mesh] OR “Carpal bones” [Mesh] OR “Radius” [Mesh] OR “Wrist” [Mesh] OR “Racial Groups” [Mesh] OR “Race factors” [Mesh] OR “White people” [Mesh] OR “Black people” [Mesh] OR “Hispanic or Latino” [Mesh] OR “Asian people” [Mesh] OR “Native Hawaiian or Other Pacific Islander” [Mesh] OR “American Indian or Alaska Native” [Mesh] OR “Pacific Island People” [Mesh] OR “Asian American Native Hawaiian and Pacific Islander” [Mesh] OR Bone Maturity [tw] OR Skeletal Maturation [tw] OR Skeletal Age [tw] OR Age Measurement [tw]
28 January 2023	Web of Science (WOS)	“Reproducibility of results” [Mesh] OR “Dimensional Measurements Accuracy” [Mesh] OR “Diagnostic Techniques and Procedures” [Mesh] OR “Diagnostic imaging” [Mesh] OR “Radiography, panoramic” [Mesh] OR “Age Determination by Skeleton” [Mesh] OR “Dentition” [Mesh] OR “Teeth” [Mesh] OR “Tooth” [Mesh] OR “Molar, Third” [Mesh] OR “Incisor” [Mesh] OR “Racial Groups” [Mesh] OR “Race factors” [Mesh] OR “White people” [Mesh] OR “Black people” [Mesh] OR “Hispanic or Latino” [Mesh] OR “Asian people” [Mesh] OR “Native Hawaiian or Other Pacific Islander” [Mesh] OR “American Indian or Alaska Native” [Mesh] OR “Pacific Island People” [Mesh] OR “Asian American Native Hawaiian and Pacific Islander” [Mesh] OR Bone Maturity [tw] OR Skeletal Maturation [tw] OR Skeletal Age [tw] OR Age Measurement [tw]

Note: (*) Search terms truncation is represented by an asterisk.

**Table 2:** Methodological quality assessment (NOS).

Authors (Yr.)	1	2	3	4	5	6	7	8	Total
Albaker et al. (2021) [46]	*	*	*	*	**		*		7
Alcina et al. (2017) [58]	*	*	*	*	*		*		6
Alshamrani et al. (2020) [55]	*	*		*	**		*		6
Alshamrani et al. (2020) [47]	*	*	*	*	*		*		6
Awais et al. (2014) [44]	*	*		*	**	*	*		7
Benjavongkulchai and Pittayapat (2018) [79]	*	*		*	*	*	*		6
Büken et al. (2007) [40]	*	*	*	*	**		*		7
Bull et al. (1999) [56]	*	*		*	*		*		5
Calfee et al. (2010) [80]	*	*		*	**	*	*		7
Cantekin et al. (2012) [41]	*	*		*	*		*		5
Chiang and Lin (2005) [52]	*	*		*	*		*		6
Dembetembe et al. (2012) [71]	*	*		*	*		*		6
Ebri (2021) [59]	*	*		*	*		*		5
Gao et al. (2022) [48]	*	*		*	**		*	*	7
Govender and Goodier (2018) [72]	*	*		*	*	*	*		6
Griffith, Cheng, and Wong (2007) [49]	*	*		*	*		*		5
Groell et al. (1999) [66]	*	*		*	**	*	*		7
Hackman and Black (2013) [57]	*	*		*	*	*	*		6
Keny et al. (2017) [35]	*	*		*	**		*		6
Kim, Lee, and Yu (2015) [50]	*	*		*	**		*		6
Kowo-Nyakoko et al. (2023) [73]	*	*	*	*	**		*	*	8
Kullman (1995) [69]	*	*		*	**		*		6
López et al. (2008) [82]	*	*	*	*	**		*		7
Magat and Ozcan (2022) [42]	*	*		*	**		*		6
Maggio, Flavel, Hart, and Franklin (2016) [76]	*	*		*	*		*		5
Mansourvar et al. (2014) [23]	*	*		*	**	*	*		7
Martinho et al. (2021) [60]	*	*	*	*	**		*		7
Martrille et al. (2023) [64]	*	*	*	*	**	*	*		8
Moradi et al. (2012) [53]	*	*		*	*	*	*		6
Mughal et al. (2014) [30]	*	*		*	*		*		5
Nang et al. (2023) [78]	*	*	*	*	**	*	*		8
Oh et al. (2012) [51]	*	*		*	**		*		6
Olaotse et al. (2023) [74]	*	*		*	*	*	*		6
Öztürk et al. (2015) [43]	*	*		*	**	*	*		7
Patel et al. (2015) [36]	*	*	*	*	**		*		7
Patil et al. (2012) [37]	*	*		*	*	*	*		6
Paxton et al. (2013) [77]	*	*		*	*	*	*		6
Pinchi et al. (2014) [62]	*	*	*	*	*	*	*		7
Pose et al. (2018) [83]	*	*		*	*		*		5
Prasad et al. (2013) [38]	*	*		*	*		*		5
Santoro et al. (2012) [63]	*	*		*	*		*		5
Santos et al. (2011) [61]	*	*	*	*	*	*	*		7
Schmidt et al. (2007) [54,67]	*	*		*	**	*	*		7
Soudack et al. (2012) [55]	*	*	*	*	*	*	*	*	7
Tineo et al. (2006) [81]	*	*	*	*	*		*		6
Tiwari et al. (2020) [39]	*	*	*	*	*	*	*		7
Tsehay et al. (2017) [75]	*	*		*	*		*		5
Van Rijn et al. (2001) [68]	*	*		*	*		*		5
Wenzel et al. (1984) [70]	*	*		*	**		*		6
Zabet et al. (2015) [65]	*	*		*	*		*		5
Zafar et al. (2010) [45]	*	*	*	*	*	*	*	*	8

Note: *Newcastle Ottawa Scale (NOS)* domains: (1) Representativeness of Exposed Cohort (*); (2) Selection of Non-Exposed Cohort (*); (3) Ascertainment of Intervention (*); (4) Demonstrate Outcome Assessed before Intervention (*); (5) Comparability of Cohorts on the Basis of Design or Analysis (**); (6) Assessment of Outcome (*); (7) Adequacy of Follow-Up (*); (8) Data available (No Missing Data) (*).

**Table 3:** Risk of bias in non-randomized studies of exposure (ROBINS-E).

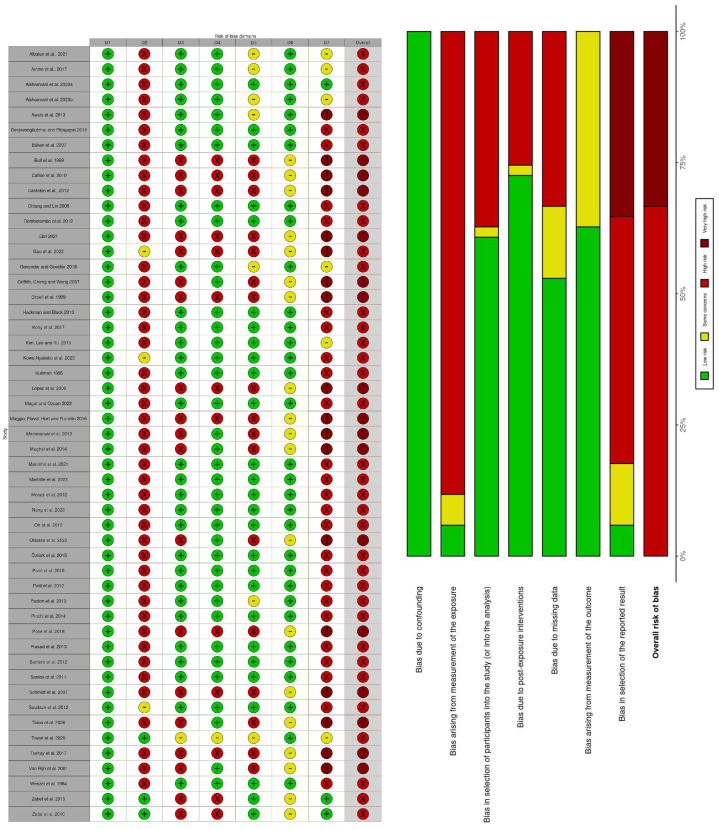

Note: *Cochrane Risk of Bias Tool for observational studies of exposures (ROBINS-E)* domains: (1) bias due to confounding; (2) bias arising from measurements of the exposure; (3) bias in selection of participants for the study (or analysis); (4) bias due to post-exposure interventions; (5) bias due to missing data; (6) bias arising from measurement of the outcome; (7) bias in selection of the reported result. In the traffic light graph the color represents the reviewer’s conclusion about the risk of each type of bias in each study being: Low risk of bias (*green*), some concerns (*yellow*), high risk of bias (*red*), very risk (*dark red*) and no information available (*blue*).

## Data Availability

The prospective review protocol is available on the International Prospective Register of Systematic Reviews PROSPERO website: https://www.crd.york.ac.uk/prospero/display_record.php?ID=CRD42023449512.

## Supplementary Materials

The following supporting information can be downloaded at: https://www.mdpi.com/article/10.3390/diagnostics13193124/s1, Table S1. Characteristics of the included studies.
