# Supplementary material for: Precision and Accuracy of Radiological Bone Age Assessment in Children among Different Ethnic Groups: A Systematic Review
Source: Diagnostics (Basel). 2023 Oct 4;13(19):3124. doi: 10.3390/diagnostics13193124 (PMC10572703; doi:10.3390/diagnostics13193124)
Supplement: Supplementary file 1 [file diagnostics-13-03124-s001.zip › diagnostics-2627110-supplementary.pdf]

diagnostics

MDPI

Authors (yr)	Study design	Outcomes	Participants	Radiographic Methods	Results
Albaker *et al.* (2021) (46)	Observational Cross-Sectional	**Precision**<br>*Repeatability*<br>*Reproducibility*	N=216, n= 68 (31.5%) females, n=148 (68.5%) males) left hand/wrist radiographs of Arab children aged 4 to 18 yrs<br>Majmaah Hospital, Saudi Arabia	**Skeletal method**<br>*GPA*	**GPA**<br>**GPA - Male**<br>• Intra-class correlation coefficient (ICC)= 0.995<br>• Correlation between CA and BA (r=0.873, p<0.001)<br>• Differences between CA and BA (MD= 143.5 mos, SD= 44.0, p<0.001)<br>**GPA - Female**<br>• Intra-class correlation coefficient (ICC)= 0.996<br>• Correlation between CA and BA (Pearson Correlation Coefficient (r) =0.872, p<0.001)<br>• Differences between CA and BA (MD= 116.9 mos, SD=41.8, p<0.001)
Alcina *et al.* (2017) (58)	Observational Retrospective Cohort	**Precision**<br>*Repeatability*<br>*Reproducibility*	N=1150, n = 560 (48.69%) females aged 0 to 18 yrs, n = 590 (51.30%) males between the ages of 0 and 19 yrs left hand/wrist radiographs of Caucasian children.<br>Faculty of Biology, University of Barcelona, Spain	**Skeletal method**<br>*GPA*	**GPA**<br>**GPA - Male**<br>• Intra-observer agreement (Cohen`s kappa coefficient) = 0.99 (p<0.05).<br>• Concordance (Lin's concordance correlation coefficient ϱc) = 0.99 (p<0.05).<br>**GPA - Female**<br>• Intra-observer agreement (Cohen`s kappa coefficient) = 0.99 (p<0.05).<br>• Concordance (Lin's concordance correlation coefficient ϱc) =0.99 (p<0.05).
Alshamrani *et al.* (2020) (55)	Observational Retrospective Cohort	**Precision**<br>*Repeatability*<br>*Reproducibility*	*GPA* (N=420, n = 200 women (47.6%), 10.21 ± 4.4 yrs, n=220 men (52.38%), 10.48 ± 4.8 yrs) left hand/wrist radiographs of Caucasians.<br>*TW3* (N=353, n = 164 women (46.45%) 8.80 ± 3.6 yrs, n=189 men (53.54%), 9.59 ± 4.4 yrs) left hand/wrist radiographs of Caucasians.<br>*BoneXpert* (N=212, n= 98 women (46.22%) 9.02 ± 3.7 yrs, n= 114 men (53.57%) 9.89 ± 3.9 yrs) left hand/wrist radiographs of Caucasians.<br>Department of Radiological Sciences, University of Najran, Saudi Arabia	**Skeletal method**<br>*GPA*<br>*TW3*<br>*BoneXpert* (automated Software)	**GPA**<br>**GPA - Male**<br>• Intra-class correlation coefficient (ICC)= 0.991<br>• GPA underestimates by 0.31 yrs/4 mos (p<0.01)<br>**GPA - Female**<br>• Intra-class correlation coefficient (ICC)= 0.984<br>**TW3 - Males**<br>• Intra-class correlation coefficient (ICC) = 0.963<br>• TW3 underestimates by 2.5 mos (p<0.01)<br>**TW3 - Females**<br>• Intra-class correlation coefficient (ICC) = 0.972<br>**BoneXpert vs GPA**<br>• GPA was lower than BoneXpert-derived G&P by an average of 0.27 yrs/3 mos in males (p<0.01) and 0.1 yrs/1 month (p=0.184) in females.<br>• GPA overestimates by 2 mos. (p=0.06) in females.<br>• GPA underestimates by 2.5 mos. (p<0.05) in males.<br>**BoneXpert vs TW3**<br>• BoneXpert overestimates by 1 mo (p<0.05) in females in comparison to TW3.<br>• BoneXpert underestimates by 2 mos (p<0.05) in males in comparison to TW3.

diagnostics MDPI

Alshamrani *et al.* (2020) (47)	Observational Retrospective Cohort	**Accuracy**	*GPA* (N= 392, n=296, Caucasians; n=71 Asians, n=20 Africans; n= 5 mixed (Caucasian/Asian) left hand/wrist radiographs.<br>*TW3* (N=353) left hand/wrist radiographs (n = 164 women (46.45%), 8.80 yrs, (SD=3.6), n=189 men (53.54%), 9.59 yrs (SD=4.4)<br>Department of Oncology and Metabolism, University of Sheffield, UK.	**Skeletal method**<br>*GPA*<br>*TW3*	**GPA**<br>**GPA-Males**<br>***T1***=3, 6 and 12 yrs old.<br>• GPA overestimated BA (p>0.05).<br>**GPA-Females**<br>***T1***=2 - 7 yrs<br>• GPA overestimated BA between 0.8 and 6 mos (p < 0.05)<br>***T2***=0 - 12 yrs old.<br>• GPA underestimated BA between 0.1 and 11 mos (p < 0.05)<br>**TW3-Females**<br>***T1***= 2- 15 yrs old.<br>• TW3 underestimated BA in 5.2 mos (p < 0.01)
Awais *et al.* (2014) (44)	Observational Cross-Sectional	**Precision**<br>*Repeatability*<br>*Reproducibility*	N=283 (n= 136 males (48.05%); 147 (51.9%) females) left hand/wrist radiographs of Caucasians.<br>Department of Radiology-Medicine, The Aga Khan University Hospital, Karachi.	**Skeletal method**<br>*GPA*<br>*Girdany and Golden (GG)*	**GPA**<br>**GPA - Male**<br>• Intra-class correlation (ICC)= 0.998<br>• Correlation between CA and SA (Pearson Correlation Coefficient (r) r=0.915.<br>**GPA - Female**<br>• Correlation between CA and SA (Pearson Correlation Coefficient (r) = 0.943.<br>**Girdany and Golden (GG) - Male**<br>• Intra-class correlation (ICC)= 0.974<br>• Correlation between CA and SA (Pearson Correlation Coefficient (r) = 0.865.<br>**Girdany and Golden (GG) - Female**<br>• Correlation between CA and SA (Pearson Correlation Coefficient (r) =0.909.
Benjavongkulchai and Pittayapat (2018) (79)	Observational Retrospective Cohort	**Precision**<br>*Repeatability*<br>*Reproducibility*<br>**Accuracy**	N=365 left hand/wrist radiographs of contemporary Thai from 6 to 20 yrs old.<br>Department of Radiology, Faculty of Dentistry, Chulalongkorn University, Bangkok, Thailand	**Skeletal method**<br>*GPA*<br>*TW3*<br>*Fishman Method*	**GPA**<br>***T1*** = 10 yrs old.<br>• Accuracy (83.2% for females and 79.63% for males)<br>• Intra-observer agreement (Cohen`s kappa coefficient) = 0.89 (p < 0.01)<br>• Inter-observer agreement (Cohen`s kappa coefficient) = 0.82 (p < 0.01)<br>**TW3 RUS**<br>• Intra-observer agreement (Cohen`s kappa coefficient) = 0.83 - 0.92 (p < 0.01)<br>• Inter-observer agreement (Cohen`s kappa coefficient) = 0.66–0.88 (p < 0.01)<br>**Fishman method**<br>***T1***= 13 - 15 yrs old.<br>• Accuracy = 77.5% for females and 74.31% for males and 83.08% for females and 73.77% for males.

					T2 = 18 yrs old.<br>• Accuracy= 53.85% for females and 54.44% for males<br>• Intra-observer agreement (Cohen`s kappa coefficient) = 0.91 (p<0.01)<br>• Inter-observer agreement (Cohen`s kappa coefficient) = 0.85 (p<0.01)
Büken *et al*. (2007) (40)	Observational Retrospective Cohort	**Accuracy**	N= 492 (n=241 (49.0%) females, n=251 (51.0%) males) left hand/wrist radiographs of Turkish Caucasians.<br>Düzce University, Düzce Medical Faculty, Forensics Medicine Department, Turkey	**Skeletal method**<br>*GPA*	**GPA - Males**<br>• Intra-observer agreement (Cohen`s kappa coefficient) = k= 0.275 (p < 0.001).<br>**GPA - Females**<br>• Intra-observer agreement (Cohen`s kappa coefficient) = k= 0.143 (p < 0.001).
Bull *et al*. (1999) (56)	Observational Prospective Cohort	**Precision**<br>*Repeatability*	N= 362 left hand/wrist radiographs of Caucasians between the ages of 2 and 18 yrs<br>Department of Radiology, Addenbrooke's Hospital, Hills Road, University of Cambridge, UK.	**Skeletal method**<br>*GPA*<br>*TW3*	**GPA**<br>• Intra-observer variation = MD=0.14 yrs, SD=1.16, 95%CI: -2.46- 2.18 (p < 0.05).<br>**TW2**<br>• Intra-observer variation MD=0.0 yrs, SD= 0.71, 95%CI:1.41- 1.43 (p < 0.05).
Calfee *et al*. (2010) (80)	Observational Prospective Cohort	**Precision**<br>*Repeatability*<br>**Accuracy**	N=138 (n=62 males (45%) and n=76 females (55%) left hand/wrist radiographs of Caucasians of 12 and 18 yrs with a minority of African American (n = 10), Hispanic (n = 2), Asian (n = 1), and other (n = 9).<br>Department of Orthopaedic Surgery, Shriners Hospital for Children, Washington University School of Medicine, United States.	**Skeletal method**<br>*GPA*	**GPA**<br>• Intra-class correlation coefficient (ICC)= 0.982<br>• Correlation between CA and BA (Pearson Correlation Coefficient (r) = 0.890 (p<0.001)
Cantekin *et al*. (2012) (41)	Observational Prospective Cohort	**Accuracy**	N= 767 (n=425 (55%) girls and n= 342 (45%) boys) left hand/wrist radiographs of Caucasian eastern Turkish children between the ages of 7 to 17 yrs<br>Department of Pedodontics, Erciyes University, Kayseri, Turkey.	**Skeletal method**<br>*GPA*	**GPA**<br>**GPA-Males**<br>• Differences between CA and BA (MD= 116.9 mos, SD=41.8, p<0.001) = -0.13 yrs (95% CI: 0.31-0.70 yrs (p>0.05)<br>***T1***=9 to 10 yrs old.<br>• GPA overestimated CA MD=0.10 yrs for 9 yrs old.<br>• GPA overestimated CA MD=0.31 yrs for 10 yrs old.<br>***T2***=10 - 17 yrs old.

					• GPA underestimated CA (MD=0.02-0.24 yrs, p<0.05)<br>**GPA-Females**<br>• Differences between CA and BA (MD= 116.9 mos, SD=41.8, p<0.001) (95%CI:0.48-0.75 yrs, p>0.05)<br>***T1***=7 – 10 yrs old.<br>• GPA underestimated CA (MD=0.40, p<0.05) for 7 yrs old.<br>• GPA underestimated CA (MD=0.48, p<0.05) for 8 yrs old.<br>• GPA underestimated CA (MD=0.11, p<0.05) for 9 yrs old.<br>• GPA underestimated CA (MD=0.24, p<0.05) for 10 yrs old.<br>***T2***=10 - 17 yrs old.<br>• GPA overestimated CA (MD=0.5, p<0.05) for 10 yrs old.<br>• GPA overestimated CA (MD=0.25, p<0.05) for 11 yrs old.<br>• GPA overestimated CA (MD=0.75, p<0.05) for 12 yrs old.<br>• GPA overestimated CA (MD=0.20, p<0.05) for 14 yrs old.<br>• GPA overestimated CA (MD=0.35, p<0.05) for 15 yrs old.<br>• GPA overestimated CA (MD=0.26, p<0.05) for 16 yrs old.<br>• GPA overestimated CA (MD=0.03, p<0.05) for 17 yrs old.
Chiang and Lin (2005) (52)	Observational Retrospective Cohort	**Precision**<br>*Repeatability*<br>*Reproducibility*<br>**Accuracy**	N=370 (n=140 (37.8%) females, n= 230 (62.2%) left hand/wrist radiographs of Taiwanese children with a mean age of 10.31 yrs Department of Radiology, Buddhist Tzu Chi General Hospital, Taiwan.	**Skeletal method**<br>*GPA*	**GPA**<br>**GPA-Male**<br>***T1***=2 - 12 yrs old.<br>• GPA underestimated CA=0.22-1.86 year (P<0.05)<br>***T2***=13 - 18 yrs old.<br>• GPA underestimated CA=0.13-1.28 year (p<0.05)<br>**GPA-Female**<br>***T1***=2 - 8 yrs old.<br>• GPA underestimated CA=0.19-0.84 mos (p<0.05)<br>***T2***=9 -17 yrs old.<br>• GPA overestimated CA=0.18-1.48 mos (p<0.05)<br>**Overall group**<br>• Inter-observer agreement (Cohen`s kappa coefficient) = 0.997 (p<0.05)
Dembetembe *et al*. (2012) (71)	Observational Prospective Cohort	**Precision**<br>*Repeatability*	N= 131 Left hand/wrist radiographs of Africans isiXhosa, is Zulu, seSotho, seTswana, and other African biological origin between the ages of 13 to 22 yrs<br>Martin Singer Cape Hand Clinic, Cape Town, South Africa.	**Skeletal method**<br>*GPA*	**GPA**<br>• Intra-observer reliability (r = 0.76, p< 0.05).<br>***T1***= 13 yrs and 18.5 yrs old.<br>• Intra-observer reliability (r= 0.02, p< 0.05).<br>***T2***=18.5 yrs and 21 yrs old.<br>• Intra-observer reliability (r = 0.55, p< 0.05).

Ebri (2021) (59)	Observational Cross-sectional study	**Accuracy**	N=160 (n=73, 45.6% males, n=87, 54.4% females) left hand/wrist radiographs of hispanic between the age of 0,5 to 20 yrs<br>Endocrinology Unit, Miguel Servet Hospital, Zaragoza, Spain.	**Skeletal method**<br>*GPA*<br>*TW2*	**GPA**<br>**GPA-Overall**<br>• GPA overestimated CA in comparison to Ebrí-carpal (EOIC) (MD=6 mos, p<0.05)<br>• GPA overestimated CA in comparison to metacarpophalangic (EOIMF) (MD=5 mos, p<0.05)<br>• GPA overestimated CA in comparison to Carpometacarpophalangic (EOICMF) (MD= to 6.5 mos, p<0.05)<br>**TW2 - Males**<br>• TW2 overestimated CA in comparison to Carpometacarpophalangic (EOICMF) (MD= 4-6 mos, p<0.05)<br>• TW2 overestimated CA from metacarpophalangic (EOIMF) and Ebrí-carpal (EOIC) (MD= 5 mos, p<0.05)<br>**TW2 - Females**<br>• TW2 overestimated CA in comparison to metacarpophalangic (EOIMF) (MD= 3 mos, p<0.05)<br>• TW2 overestimated CA in comparison to EO Ebrí-carpal (EOIC) (MD= 4 mos, p<0.05)<br>• TW2 overestimated CA in comparison to Carpometacarpophalangic (EOICMF) (MD= 4-7 mos, p<0.05
Gao *et al.* (2022) (48)	Observational Retrospective Cohort	**Accuracy**	N=390 (n=207 females (53.07%); n=187 males (56.93%) Left hand/wrist radiographs of Asiatic Zhejiang Province aged between 3 to 6 yrs<br>Department of Radiology, Affiliated Hangzhou First People's Hospital, Zhejiang University School<br>of Medicine, Hangzhou, China.	**Skeletal method**<br>*GPA*<br>*TW3*<br>*RUS–CHN (China 05)*	**GPA - Males**<br>• Accuracy=12.02%<br>**GPA - Females**<br>• Accuracy=25.76%<br>**TW3- Males**<br>• Accuracy=32.24%<br>**TW3 - Females**<br>• Accuracy=24.15%<br>**RUS–CHN (China 05) – Males**<br>• Accuracy= 12.02%<br>**RUS–CHN (China 05) - Females**<br>• Accuracy=21.26%
Govender and Goodier (2018) (72)	Observational Cross-sectional study	**Precision**<br>*Repeatability*<br>**Accuracy**	N=102 Left hand/wrist radiographs of both sexes, aged between 0 to 21 yrs<br>KwaZulu-Natal, Grey's Hospital, Pietermaritzburg,<br>South Africa	**Skeletal method**<br>*GPA*	**GPA**<br>• Intraclass coefficient (ICC) = 0.99 (95% CI, p<0.001)<br>• Differences between CA and BA (MD = 7.4 ± 15.7 mos (95% CI, p<0.05)<br>**GPA - Males**<br>*T1*=≤19 yrs old.<br>• Differences between CA and BA (MD = 4.4 ± 14.5 mos, 95% CI, p<0.05).<br>• GPA overestimated CA (MD=3 ± 5 mos, p<0.05)<br>**GPA - Females**

					T1=≤18 yrs old.<br>• Differences between CA and BA (MD = 2.4 ± 12.8 mos, 95% CI, p<0.05).<br>• GPA overestimated CA (MD= 1.8 ± 20.2, p<0.05).
Griffith, Cheng, and Wong (2007) (49)	Observational Retrospective cohort	**Accuracy**	N= 1016 (female n= 366 (36.2%); male n= 650 (63.8%) left hand/wrist radiographs of Chinese aged 18 yrs or less.<br>Accident and emergency departments of Prince of Wales North District, and Tuen Mun hospitals, China.	**Skeletal method**<br>*GPA*<br>*TW3*	**GPA vs TW3 RUS - Females**<br>***T1***=0 - 5.5 yrs old.<br>• Accuracy = TW3 RUS method underestimates CA in comparison to GPA (p<0.0001)<br>***T2***=4.5 - 11.5 yrs old.<br>• TW3 RUS method overestimated CA in comparison to GPA (p<0.0001)<br>***T3***= ≥ 16 yrs old.<br>• TW3 RUS method overestimated CA in comparison to GPA (p<0.0001)<br>**GPA vs TW3 RUS - Males**<br>***T1***= 5 - 9 yrs old.<br>• TW3 RUS method underestimated CA in comparison to GPA (p<0.0001)<br>***T2***= ≥ 8 yrs old.<br>• TW3 RUS methods were significantly more accurate in comparison to GPA (p<0.0001)<br>***T3***= 6 - 18 yrs old.<br>• TW3 RUS methods overestimated CA in comparison to GPA (p<0.0001)
Groell *et al.* (1999) (66)	Observational Retrospective cohort	**Accuracy**	N= 47 children (n=21, 44.7% females, n=26, 55.3% males) left hand/wrist radiographs of Caucasians aged ranging from 2 mos to 18.8 yrs, with a mean age of 8.7 yrs<br>Division of Pediatric Radiology, Department of Radiology and Department of Pediatric Surgery, University Hospital Graz, Graz, Austria	**Skeletal method**<br>*GPA*	**GPA**<br>• Differences between CA and BA (MD = 95% CI=0.4±4.0 vs -1.1± 5.9 mos, p=0.20)<br>• Differences between CA and BA (MD = 95% CI=1.5±7.6, p=0.20) for staff radiologists.<br>• Differences between CA and BA (MD = 95% CI= 2.7±10.3, p=0.09) for residents.
Hackman and Black (2013) (57)	Observational Retrospective cohort	**Precision**<br>*Repeatability*<br>**Accuracy**	N=406 (n=157 (38.7%) females; n=249 (61.3%) males) left hand/wrist radiographs of Caucasians between the ages of birth and 21 yrs of age.<br>Accident and Emergency Department of Ninewells Hospital in Dundee, Scotland, United Kingdom.	**Skeletal method**<br>*GPA*	**GPA**<br>**GPA – Males**<br>• Intra-observer reliability (Determination coefficient (r^2) = 0.940, p < 0.001.<br>• Differences between CA and BA (MD=1.63 mos, SD=14.16, p=0.899)<br>***T1***= 0 - 2 yrs old.<br>• GPA underestimated (MD=0.2-10 mos, p>0.05)<br>***T2***= 0 - 10 yrs old.

					• GPA underestimated CA (MD=2.44 - 3.54 mos, p>0.05)<br>***T3*** = 11 - 15 yrs old.<br>• GPA overestimated CA (MD=1.74 mos, p<0.05)<br>***T4*** = 13 - 17 yrs old.<br>• GPA overestimated CA (MD=1.62 to 11.05 mos, p<0.05)<br>**GPA – Females**<br>• Intra-observer reliability (Determination coefficient (r^2) =0.939, p < 0.001).<br>• Differences between CA and BA (MD = 1.95 mos, SD=14.97, p = 0.771)<br>***T1*** = ≤9 yrs old.<br>• GPA overestimated CA (MD=1.14 – 5.12 mos, p<0.05)<br>***T2*** = 9-17 yrs old.<br>• GPA overestimated CA (MD=0.20 – 5.73 mos, p<0.05)<br>***T3*** = 0-15 yrs old.<br>• GPA overestimated CA/ MD=2.04 – 3.06 mos, p<0.05)
Keny *et al.* (2017) (35)	Observational Retrospective cohort	**Precision**<br>*Repeatability*<br>**Accuracy**	N=106 (n=51 females (48.1%), 55 males (51.8%) left hand/wrist radiographs of indian children between the ages of 1 and 15 yrs Department of Orthopaedics, Gokuldas Tejpal Hospital, Mumbai, India.	**Skeletal method**<br>*GPA*<br>*MacKay's* (MK) method	**GPA**<br>**GPA Overall Group**<br>• Inter-rater reliability (Cohen`s kappa coefficient) = 0.68 (95%CI= 0.504–0.848, (p< 0.001).<br>**GPA – Males**<br>• Differences between CA and BA (MD= 10 mos, p<0.05)<br>• Repeatability = 44%<br>***T1*** = 1-6 yrs old.<br>• GPA overestimated CA (MD= 10 mos, p<0.05)<br>**GPA - Females**<br>• Differences between CA and BA (MD= 8 mos, p<0.05)<br>• Reliability=25%<br>**T1** = 1-6 yrs old.<br>• GPA overestimated CA (MD= 8 mos, p<0.05)<br>**MacKay's (MK) - Males**<br>• Differences between CA and BA (MD= 22 mos, p<0.05)<br>• Reliability=10%<br>***T1*** = 1-6 yrs old.<br>• GPA overestimated CA (MD= 23 mos, p<0.05)<br>**MacKay's (MK) - Females**<br>• Differences between CA and BA (MD= 17 mos, p<0.05)<br>• Reliability=16%<br>***T1*** = 1-6 yrs old.<br>• GPA overestimated CA (MD= 17 mos, p<0.05)
Kim, Lee and Yu (2015) (50)	Observational	**Precision**<br>*Repeatability*	N=212 (n= 77 females (36.3%), n= 135 males (63.7%) left hand/wrist	**Skeletal method**<br>*GPA*	**GPA**

	Retrospective cohort	*Reproducibility*	radiographs of Korean children aged between 7 and 12 yrs Department of Radiology and Pediatrics, Dankook University Hospital, Cheonan, South Korea.	TW3<br>KS (Korean Standard Bone age chart)	• Intra-observer coefficient (Determination coefficient (r^2) = 0.9, $p < 0.001$).<br>• Differences between CA and BA (MD=0.45 mos, SD= 1.79 mos)<br>**TW3**<br>• Intra-observer coefficient (Determination coefficient (r^2) = 0.88, $p < 0.001$).<br>• Differences between CA and BA (MD=0.45 mos, SD= 1.81 mos)<br>**KS (Korean Standard Bone age chart)**<br>• Intra-observer coefficient (Determination coefficient (r^2) = 0.89, $p<0.001$).<br>• Differences between CA and BA (MD=0.21 mos, SD= 1.19 mos, $p<0.05$)<br>**Overall Group**<br>• Inter-observer coefficient (Pearson's correlation coefficient r= 0.95 (95%CI = 0.996-1.000, $p<0.05$).<br>• Difference between three methods (F ratio= 0.10, p=0.9).
Kowo-Nyakoko *et al.* (2023) (73)	Observational Cross-sectional study	**Precision**<br>*Repeatability*<br>*Reproducibility*<br>**Accuracy**	N=252 (n=111 (44%) females, n= 141 (56 % males) left hand/wrist radiographs of peripubertal children in Zimbabwe.<br>MRC Lifecourse Epidemiology Centre, University of Southampton, Southampton General Hospital, Southampton, United Kingdom.	**Skeletal method**<br>*GPA*<br>TW3	**GPA**<br>• Intraclass correlation coefficient (ICC) = 0.94<br>• Intra-rater reliability = 0.98<br>• Mean percentage coefficient of variation (Inter-operator 1.5% vs 3.7%)<br>**GPA-Males**<br>• Intra-observer reliability (Pearson's determination coefficient r=0.93 ($p< 0.05$).<br>• Differences between CA and BA (MD= 0.76 yrs, 95%CI: −0.95, −0.57, $p<0.05$)<br>**GPA and TW3 - Males**<br>• Correlation between CA and BA= GPA β 1.09 (CI 95 %: 1.01, 1.17); TW3 β=0.95 (95 % CI: 0.87-1.03, $p<0.05$).<br>**GPA - Females**<br>• Intra-observer reliability (Pearson's determination coefficient r=0.92, $p<0.05$).<br>**GPA and TW3 - Females**<br>• Correlation between CA and BA= GPA β 1.09 (95 % CI: 0.98, 1.20); TW3 β= 0.92 (95 % CI: 0.81, 1.03).<br>**TW3**<br>**TW3 (RUS) - Males**<br>• Intra-observer reliability (Determination coefficient (r2) = 0.91 ($p<0.05$).<br>• Differences between CA and BA (MD=-0.43 years, 95%CI: -0.61,-0.24, $p<0.05$)<br>**TW3 (RUS) - Females**

					• Intra-observer reliability (Pearson's determination coefficient (r^2) = 0.88 ($p<0.05$).<br>**TW3 (RUS) Group**<br>• Intraclass correlation coefficient (ICC) = 0.98<br>• Intra-rater reliability= 0.97<br>• Mean percentage coefficient of variation (Intra-operator 1.5 % vs 2.4 %)
Kullman (1995) (69)	Observational Retrospective cohort	**Precision**<br>*Repeatability*<br>**Accuracy**	N=72 left hand/wrist radiographs and OPG of Caucasian Swedish adolescents aged between 12 to 19 yrs from Stockholm, Malmö and Örebro.<br>Department of Oral Diagnosis, Oral Radiology and Forensic Odontology, Karolinska Institute, Stockholm. Sweden	**Skeletal method**<br>*GPA*<br>**Dental method**<br>(Lengths of the roots of the lower third molars and total tooth lengths following Kullman et al., (1995) method)	**GPA**<br>• Intra-observer reliability (r=0.64-0.74)<br>**GPA - Male**<br>• Differences between CA and BA (MD=0.4 yrs, $p>0.05$)<br>**GPA - Female**<br>• Differences between CA and BA (MD=0.4 yrs, $p>0.05$)<br>**Dental method - Male**<br>• Subjectively estimated root development (MD=1.2 yrs, SD=1.0-1.4, $p<0.05$)<br>• Digitized relative root length (MD =1.5 yrs, SD =1.0-1.4, $p<0.05$)
López *et al*. (2008) (82)	Observational Cross-sectional study	**Accuracy**	N=160 left hand/wrist radiographs of Hispanics from Venezuela aged from 7 to 14 yrs<br>Colegios Cardonal Wayúu y La Resistencia, Zulia, Venezuela	**Skeletal method**<br>*TW3 RUS*<br>*TW3 Carpal*	**TW3**<br>**TW3 RUS - Male**<br>• Correlation between CA and BA (Pearson Correlation Coefficient (r) = 0.91 ($p < 0.05$).<br>**TW3 RUS - Female**<br>• Correlation between CA and BA (Pearson Correlation Coefficient (r) =0.93 ($p < 0.05$).<br>**TW3 Carpal - Male**<br>• Correlation between CA and BA (Pearson Correlation Coefficient (r) =0.89 ($p < 0.05$).<br>**TW3 Carpal - Female**<br>• Correlation between CA and BA (Pearson Correlation Coefficient (r) = 0.82 ($p < 0.05$).
Magat and Ozcan (2022) (42)	Observational Retrospective cohort	**Precision**<br>*Repeatability*<br>*Reproducibility*	N= 284 (n= 176 females (61.9%) 13.96±1.86 yrs, n=108 males (38.1%), 14.26±1.96 yrs) left hand/wrist radiograph and panoramic, lateral cephalometric of Turkish adolescent aged 9 to 19 yrs<br>Department of Oral and Maxillofacial Radiology, Faculty of Dentistry, Necmettin Erbakan University, Konya, Turkey.	**Skeletal method**<br>*CVM* (cervical vertebrae maturation following Baccetti et al. (2002) method)<br>*HWM* (Hand and wrist maturation- Ru stage based on Skeletal maturity indicators)	**CVM**<br>• Inter-observer reliability (Cohen`s kappa coefficient) = 0.862 - 0.958.<br>**HWM**<br>• Inter-observer reliability (Cohen`s kappa coefficient) = 0.812 - 0.961.<br>• Intra-observer reliability HWM and CVM stages (Pearson Correlation Coefficient (r)=0.809 ($p<0.05$)<br>**DM**<br>• Inter-observer reliability (Cohen`s kappa coefficient) =0.823 - 0.928.<br>• Intra-observer reliability DM and HWM stages (Pearson Correlation Coefficient (r)=0.561 ($p<0.05$)

				**Dental method**<br>*DM* (Demirjian's classification system)	
Maggio, Flavel, Hart and Franklin (2016) (76)	Observational Retrospective cohort	**Precision**<br>*Reproducibility*<br>**Accuracy**	N= 360 left hand/wrist radiograph Caucasian Western Australians (Perth region)<br>Centre for Forensic Anthropology, School of Anatomy, Physiology and Human Biology, The University of Western Australia, Crawley, Australia	**Skeletal method**<br>*GPA*	**GPA**<br>• Inter-rater agreement (Cohen`s kappa coefficient) =0.887 ($p<0.001$)<br>**GPA - Male**<br>• Estimation range= 0.0–19.0 yrs (SD=± 6.0 yrs)<br>• Standard error of the estimate (SEE)= ±0.005 yrs<br>• Correlation between CA and BA (Pearson Correlation Coefficient (r)= 0.970 ($p<0.05$)<br>**GPA - Female**<br>• Estimation range= 0.0–18.0 yrs (SD ±5.8 yrs).<br>• Standard error of the estimate (SEE)= ±0.412 yrs<br>• Correlation between CA and BA (Pearson Correlation Coefficient (r)= 0.972 ($p<0.05$)
Mansourvar *et al.* (2014) (23)	Observational Retrospective cohort	**Accuracy**	N=184 (n=48, Asian (1-8 yrs), n= 47 African/American (8-15 yrs), n= 46 Caucasian (10-16 yrs), n= 43 Hispanic (15-18) left hand/wrist radiographs of Children's Hospital Los Angeles (CHLA).<br>Faculty of Medicine, University of Malaya (UM), Kuala Lumpur, Malaysia.	**Skeletal method**<br>*GPA*	**GPA - Asian**<br>***T1***= 4 yrs<br>• Differences between CA and BA (MD =2.3 yrs, $p>0.05$)<br>**GPA - African/American**<br>***T1***= 15 yrs<br>• Differences between CA and BA (MD =2.4 yrs, $p>0.05$)<br>**GPA - Caucasian**<br>***T1***= 10-16 yrs<br>• Differences between CA and BA (MD =0.044 yrs, $p>0.05$)<br>**GPA - Hispanic**<br>***T1***= 15-18 yrs<br>• Differences between CA and BA (MD =0.094 yrs, $p>0.05$)
Martinho *et al.* (2021) (60)	Observational Cross-sectional study	**Precision**<br>*Repeatability*<br>*Reproducibility*	N=441 (100%) left hand/wrist radiographs of Portuguese female soccer players 10.08 to 16.73 yrs of age (14.38 ± 1.24 yrs).<br>University of Coimbra, FCDEF, Coimbra, Portugal	**Skeletal method**<br>*GPA*<br>*FELS*	**GPA vs FELS**<br>• Intraindividual differences = 0.10 - 1.47 yrs ($p<0.05$) in BA.<br>***T1***=< 13 yrs<br>• Inter-rater agreement (Cohen`s kappa coefficient) = 0.48 ($p>0.05$).<br>***T2***=< 14 yrs<br>• Inter-rater agreement (Cohen`s kappa coefficient) = 0.39 ($p<0.05$).<br>• Concordance (Lin's concordance correlation coefficient ϱc) = 0.68 ($p<0.05$).<br>***T3***=< 17 yrs<br>• Inter-rater agreement (Cohen`s kappa coefficient) = 0.01 ($p>0.05$)<br>• Concordance (Lin's concordance correlation coefficient ϱc) = 0.11 ($p<0.05$).
Martrille *et al.* (2023) (64)	Observational	**Precision**<br>*Repeatability*	N=94 (n=64 males (68.1%), n=30 females (31.9%) left hand/wrist	**Skeletal method**<br>*GPA*	**GPA**<br>• Intraclass correlation coefficient (ICC) = 0.94 (95% CI=0.91-0.96)

	Retrospective cohort	**Accuracy**	radiographs of French children's hospital with the suspicion of child physical abuse/maltreatment. Montpellier University Hospital, France.	**Dental method** *Anderson, Demirjian, Schour and Massler, and Ubelaker revised method, Gustafson and Koch.*	• Differences between CA and BA (MD =1.27, SD= 1.56 mos, p<0.05).<br>**Anderson**<br>• Intraclass correlation coefficient (ICC) = 0.88 (95% CI= 0.68-0.96).<br>• Differences between CA and BA (MD =5.12, SD= 1.41 mos, p<0.05).<br>**Demirjian**<br>• Intraclass correlation coefficient (ICC) = 0.85 (95%CI= 0.50-0.96)<br>• Differences between CA and BA (MD =6.47, SD= 1.99 mos, p<0.05).<br>**Schour and Massler**<br>• Intraclass correlation coefficient (ICC) = 0.98 (95%CI= 0.98-0.99)<br>• Differences between CA and BA (MD =1.45, SD= 1.99 mos, p<0.05).<br>**Ubelaker**<br>• Intraclass correlation coefficient (ICC) = 0.97 (95%CI = 0.97-0.99)<br>• Differences between CA and BA (MD =1.37, SD= 1.83 mos, p<0.05).<br>**Gustafson and Koch Method**<br>• Intraclass correlation coefficient (ICC) = 0.98 (95%CI= 0.97-0.98)<br>• Differences between CA and BA (MD =1.50, SD= 1.99 mos, p<0.05).
Moradi *et al.* (2012) (53)	Observational Cross-sectional study	**Accuracy**	N=425 (n=122 female (28.7%; n=303 male (71.3%) left hand/wrist radiographs of healthy Arab children aged between 6 and 18 yrs General Hospital, Isfahan, Iran	**Skeletal method** *GPA*	**GPA**<br>• Differences between CA and BA (MD =0.25 yrs, SD= 0.95 yrs, p>0.05).<br>**GPA-Males**<br>• Differences between CA and BA (MD = 0.37 yrs, SD= 0.98 yrs, p>0.05)<br>**GPA-Females**<br>• Differences between CA and BA (MD = 0.04 yrs, SD= 0.78 yrs, p>0.05)
Mughal *et al.* (2014) (30)	Observational Cross-sectional study	**Precision** *Reproducibility*	N=220 (n=81 females (36.8%); n=139 males (63.2%) left hand/wrist radiographs of Arab children between ages of 6.65 ± 13.47 mos in females and 15.78 ± 12.83 mos in males. Ziauddin Hospital Clifton, Karachi, Pakistan	**Skeletal method** *GPA*	**GPA**<br>**GPA-Males**<br>• Correlation between CA and BA (Pearson Correlation Coefficient (r)= 0.778 (p<0.001)<br>**GPA-Females**<br>• Correlation between CA and BA (Pearson Correlation Coefficient (r)=0.778 (p<0.001)
Nang *et al.* (2023) (78)	Observational Cross-sectional study	**Precision** *Repeatability* *Reproducibility* **Accuracy**	N=182 (n=103 females (56.6%), n=79 (43.4%) males) left hand/wrist radiographs of children between the ages of 9 to 18 yrs Department of Pathology and Microbiology, Faculty of Medicine & Health Sciences, University Malaysia Sabah, Kota Kinabalu, Malaysia.	**Skeletal method** *GPA*	**GPA**<br>**GPA-Male**<br>• Intraclass correlation coefficient (ICC) = 0.947 (p=0.86)<br>• Correlation between CA and BA (Pearson Correlation Coefficient (r) = 0.81(p<0.01)<br>• GPA underestimated CA (MD=0.6 yrs, (p<0.05)<br>**GPA-Female**<br>• Intraclass correlation coefficient (ICC) = 0.93 (p=0.33).<br>• Correlation between CA and BA (Pearson Correlation Coefficient (r) = 0.723 (p<0.01)<br>• GPA underestimated CA (MD= 0.7 yrs, (p<0.05)<br>**Overall group by time**

					• Intraclass correlation coefficient (ICC) = 0.937<br>• Inter-rater reliability F ratio = 0.804 (p = 0.37)<br>***T1***=13.0–13.9 yrs old.<br>• Differences between CA and BA (MD =−0.6 yrs, p<0.05).<br>***T2***=17.0–17.9 yrs old.<br>• Differences between CA and BA (MD =−0.8 yrs, p<0.05).<br>***T3***=18.0–18.9 yrs old.<br>• Differences between CA and BA (MD =−1.5 yrs, p<0.05).
Oh *et al.* (2012) (51)	Observational Cross-sectional study	**Accuracy**	N=378 (n=237 girls (62.7%), n=141 (37.30%) left hand/wrist radiographs of Asiatic healthy children in puberty.<br>Ewha Womans University Mokdong<br>Hospital, Seoul, South Korea	**Skeletal method**<br>*GPA*<br>*TW3*	**GPA**<br>• Differences between CA and BA (11.4 ± 2.2 yrs vs 11.8 ± 2.5, p< 0.05)<br>**GPA - Males**<br>• GPA overestimated 54.6% in CA (p< 0.05).<br>**GPA - Females**<br>• GPA overestimated 74.3% in CA (p<0.05).<br>**TW3**<br>• Differences between CA and BA (11.9 ± 2.8 yrs vs 11.8 ± 2.5, p<0.05)<br>**TW3-Male**<br>• GPA overestimates 59.6% in CA (p<0.05).<br>**TW3-Female**<br>• GPA overestimates 72.2 % in CA (p<0.05).
Olaotse *et al.* (2023) (74)	Observational Cross-sectional study	**Precision**<br>*Repeatability*<br>*Reproducibility*<br>**Accuracy**	N=140 (n=60, 42.8% females, n=80, 57.2% males) left hand/wrist radiographs of African children aged between 5 and 18 yrs<br>Department of Chemical and Forensic Sciences, Botswana International University of Science and Technology, Palapye, Botswana.	**Skeletal method**<br>*GPA*	**GPA-Males**<br>• Intra-class correlation coefficient = 0.97 (p>0.05)<br>• Inter-observer analysis = 0.93 (p>0.05)<br>• Correlation between CA and BA (Pearson's determination coefficient (r^2) = 0.87, p<0.05.<br>• GPA overestimated by up to 4 yrs (p<0.05)<br>• GPA underestimates by up to 5 yrs (p<0.05)<br>**GPA - Female**<br>• Intra-class correlation coefficient = 0.98 (p>0.05)<br>• Inter-observer analysis = 0.94 (p>0.05)<br>• Correlation between CA and BA (Pearson's determination coefficient (r^2) = 0.85 (p < 0.05)<br>• Differences between CA and BA (− 4, - 5 yrs, p< 0.05)<br>• GPA overestimated by up to 4 yrs (p<0.05)<br>• GPA underestimated by up to 7 yrs (p<0.05)<br>**Overall group by time**<br>***T1***=5 ≤ 10 yrs old.<br>• Differences between CA and BA (MD =0.25 yrs, p<0.05).<br>***T2***=10 ≤ 15 yrs old.<br>• Differences between CA and BA (MD =0.48 yrs, p<0.05).<br>***T3***=15 ≤ 18 yrs old.<br>• Differences between CA and BA (MD =0.94 yrs, p<0.05).

Öztürk *et al.* (2015) (43)	Observational Retrospective cohort	**Accuracy**	N=849 (n= 514 girls (60.5%), n=375 boys (44.1%) left hand/wrist radiographs of Central Anatolians aged between 9 and 17 yrs<br>N= 414 Eastern Anatolian Turkish Caucasian children aged between 9 and 17 yrs<br>Department of Orthodontics of Cumhuriyet University, Turkey. Faculty of Dentistry in Central Anatolia and Inonu University Eastern Anatolia, Turkey.	**Skeletal method**<br>*GPA*	**GPA**<br>**GPA-Males**<br>• Differences between CA and BA in Malatya and Sivas (MD=–1.19, 95%CI: 12.81 ± 2.3, 13.71 ± 2.6 yrs, $p < 0.05$).<br>**GPA-Females**<br>• Differences between CA and BA in Malatya and Sivas (MD=–0.90, 95%CI: 12.91 ± 2.3, 14.11 ± 2.6 yrs, $p<0.05$).<br>***T1***= 10-13 yrs old.<br>• Differences between CA and BA in Malatya ($p<0.05$).<br>***T2***= 9, 11, 12, 14, 15 and 16 yrs old.<br>• Differences between CA and BA in Siva ($p>0.05$).
Patel *et al.* (2015) (36)	Observational Cross-sectional study	**Precision**<br>*Reproducibility*<br>**Accuracy**	N=180 (90 females (50%), 90 males (50%) ranging from 6 to 16 yrs of age of left hand/wrist radiographs and orthopantomography (OPG) images of left quadrant mandibular teeth.<br>Gandhinagar, India.	**Skeletal method**<br>*GPA*<br>**Dental method**<br><br>DM (Demirjian's classification system)<br>Willem's method	**GPA**<br>**GPA -Male**<br>• Accuracy of predicting age= 90.65% ($p>0.05$)<br>• Correlation between CA and BA ($r=0.921$, $p < 0.001$)<br>**GPA - Female**<br>• Accuracy of predicting age= 89.04% ($p>0.05$)<br>• Correlation between CA and BA ($r=0.960$, $p < 0.001$)<br>**Demirjian's classification system-Male**<br>• Accuracy of predicting age= 86.73% ($p>0.05$)<br>• Correlation between CA and BA ($r=0.882$, $p < 0.001$)<br>***T1***=6-10.99 yrs old.<br>• Dental Age overestimated CA ($p>0.05$)<br>***T2***=11-14.99 yrs old.<br>• Dental Age underestimated CA ($p>0.05$)<br>**Demirjian's classification system-Female**<br>• Correlation between CA and BA ($r=0.956$, $p < 0.001$)<br>**Willem's method - Male**<br>• Correlation between CA and BA ($r=0.921$, $p < p>0.05$)<br>**Willem's method - Female**<br>• Accuracy of predicting age= 89.08%% ($p>0.05$)<br>• Correlation between CA and BA (Pearson Correlation Coefficient (r)=0.959 ($p>0.05$)<br>**Overall group**<br>• Intra-observer variability (Pearson's Chi-square test) =0.164 ($p>0.05$).
Patil *et al.* (2012) (37)	Observational Cross-sectional study	**Accuracy**	N=375 (n=181(48.2%) females, n=194 males (51.7%) left hand/wrist radiographs of Indian children aged 1 day to 19 yrs	**Skeletal method**<br>*GPA*	**GPA**<br>**GPA - Males**<br>***T1***= 8–9 yrs old.<br>• Differences between CA and BA (MD = 2.11 yrs, $p<0.05$).<br>***T2***= 8–13 yrs old.<br>• Differences between CA and BA (MD = 1.33 yrs, $p<0.05$).

			Department of Anatomy, Government Medical College, Maharashtra, India		**GPA - Females**<br>***T1***= 0–18 yrs old.<br>• Differences between CA and BA (MD = 0.2 – 0.8 yrs, p<0.05).<br>***T2*** = 4–8 yrs old.<br>• Differences between CA and BA (MD = 0.52 yrs, p<0.05).<br>***T3*** = 19 yrs old.<br>• Differences between CA and BA (MD = 0.22 yrs, p<0.05).
Paxton *et al.* (2013) (77)	Observational Cross-sectional study	**Precision**<br>*Repeatability*<br>**Accuracy**	N=406 (n=130 females (32.0%), n=276 males (68.0%) left hand/wrist radiographs of Caucasian children underwent hand X-rays.<br>The Townsville Hospital, Queensland, Australia.	**Skeletal method**<br>*GPA*	**GPA**<br>• Differences between CA and BA (MD = -2.2 mos, p=0.005).<br>**GPA-Males**<br>• Differences between CA and BA (MD = 1.5 mos, p= 0.142).<br>**GPA-Females**<br>• Differences between CA and BA (MD = 3.7 mos, p=0.002).<br>**Overall group by time**<br>***T1***= Early childhood<br>• Differences between CA and BA (MD = 0.81 mos, p=0.719).<br>***T2***= Adolescence<br>• Differences between CA and BA (MD = -3.8 mos, p=0.001).<br>• Intra-observer variability (Pearson's Chi-square test) = -0.29 mos (p=0.846).
Pinchi *et al.* (2014) (62)	Observational Retrospective cohort	**Precision**<br>*Repeatability*<br>*Reproducibility*<br>**Accuracy**<br>**Sensitivity**<br>**Specificity**	N=307 (n=145 females (47.2%), n=162 males (52.8%) left hand/wrist radiographs of Italian children or adolescents aged between 6 and 20 yrs<br>Department of Health Sciences, Forensic Sciences Section, University of Florence, Florence, Italy.	**Skeletal method**<br>*GPA*<br>*TW2*<br>*TW3*	**GPA**<br>**GPA-Males**<br>• Intra-rater reliability (Pearson' correlation coefficient) r =0.907 (95%CI= 0.761-0.966, p<0.05)<br>• Sensitivity (%) = 90.0<br>• Specificity (%) = 87.18<br>• Accuracy (%) 88.14<br>**GPA-Females**<br>• Intra-rater reliability (Pearson' correlation coefficient) r =0.928 (95% CI= 0.789-0.977, p<0.05)<br>• Sensitivity (%) = 85.71<br>• Specificity (%) = 82.76<br>• Accuracy (%) = 83.33<br>**TW2 -Males**<br>• Intra-rater reliability (Pearson' correlation coefficient) r = 0.862 (95% CI= 0.759-0.949, p<0.05)<br>• Sensitivity (%) = 100.0<br>• Specificity (%) = 72.92<br>• Accuracy (%) = 80.88<br>**TW2 -Females**<br>• Intra-rater reliability (Pearson' correlation coefficient) r = 0.929 (95% CI= 0.793-0.978, p<0.05)

					• Sensitivity (%) = 87.50<br>• Specificity (%) =72.41<br>• Accuracy (%) = 75.67<br>**TW3 -Males**<br>• Intra-rater reliability (Pearson' correlation coefficient) r = 0.843 (95%CI= 0.617-0.942, p<0.05)<br>• Sensitivity (%) = 90.0<br>• Specificity (%) =87.5<br>• Accuracy (%) =88.24<br>**TW3 -Females**<br>• Intra-rater reliability (Pearson' correlation coefficient) r = 0.910 (95%CI= 0.817-0.956, p<0.05)<br>• Sensitivity (%) = 71.42<br>• Specificity (%) = 83.87<br>• Accuracy (%) = 81.57
Pose *et al.* (2018) (83)	Observational Retrospective cohort	**Precision**<br>*Reproducibility*<br>**Accuracy**	N=1493 (n=922 females (61.8%), n=571 (38.2%) males left hand/wrist radiographs of Hispanic children unde16 yrs of age median chronological age of 9.96 yrs and 11.12 yrs for males (p 0.001). Clínica Alemana de Santiago, Santiago, Chile.	**Skeletal method**<br>*GPA*<br>BoneXpert® (Automated Software)	**GPA vs BoneXpert® (Automated Software)**<br>• Correlation between CA and BA (Pearson' correlation coefficient (r) = 0.91- 0.93 (p<0.05)<br>• Difference between manual BA and BoneXpert® (MD=0.19 yrs, 95%CI: 0.13-0.25, p<0.05)
Prasad *et al.* (2013) (38)	Observational Cross-sectional study	**Accuracy**	N=50 (n=50 females (100%) left hand/wrist and lateral cervical radiographs of Indian in the age group of 8 to 14 yrs of age. Department of Orthodontics, Army College of Dental Sciences, Secunderabad, Andhra Pradesh, India.	**Skeletal method**<br>*CVM* (cervical vertebrae maturation)	**CVM (cervical vertebrae maturation)**<br>• Correlation between VA and BA (Pearson' correlation coefficient (r) = 0.915 (p=0.000)<br>• Correlation between VA and CA (Pearson' correlation coefficient (r) = 0.797 (p=0.000)<br>• Correlation between CA and BA (Pearson' correlation coefficient (r) = 0.844 (p=0.000)<br>• Differences between VA and BA (MD = 0.170 ± 1.08 yrs (p>0.05).<br>• Differences between VA and CA (MD = 0.097 ± 0.793 year (p>0.05).
Santoro *et al.* (2012) (63)	Observational Retrospective cohort	**Accuracy**	N=535 (n=92 female (54.65%), 243 male (45.4%) left hand/wrist radiograph and OPG of children between the ages of 7 and 15 yrs (Mean = 10.4, SE = 0.07 yrs) Department of Legal Medicine, University of Bari, Bari, Italy	**Skeletal method**<br>*GPA*<br>**Dental method**<br>DM (Demirjian's classification system)	**GPA**<br>**GPA -Male**<br>• Differences between CA and BA (MD = 0.1 yrs, SD=1.3; t = 1.31; p = 0.18).<br>• Correlation between CA and BA (Determination coefficient (r2) = 0.78 (p< 0.0001).<br>**GPA -Female**<br>• Differences between CA and BA (MD =0.4 yrs, SD=1.0; t = 5.96; p < 0.0001).

					• Correlation between CA and BA (Determination coefficient (r2) = 0.67 (p< 0.0001).<br>**Demirjian's classification system**<br>• Correlation between DA and BA (Determination coefficient (r^2) = 0.60 (p < 0.0001).<br>**Demirjian's classification system - Male**<br>• Differences between DA vs CA (MD = (1.0 yrs, SD=1.5; t = 10.52; p < 0.0001).<br>**Demirjian's classification system - Female**<br>• Differences between DA vs CA (MD = 1.1yrs, SD=1.6; t = 11.56; p < 0.0001).
Santos *et al.* (2011) (61)	Observational Retrospective cohort	**Precision**<br>*Repeatability*<br>*Reproducibility*<br>**Accuracy**	N=230 left hand/wrist radiograph from Portuguese children aged between 12 and 20 yrs<br>University of Coimbra, Coimbra, Portugal	**Skeletal method**<br>*GPA*<br>*MT* (Maturo's method)	**GPA**<br>**GPA -Male**<br>• Intra-observer (Pearson's correlation coefficient) r=0.99 (p<0.05)<br>• Inter-observer (Pearson's correlation coefficients) r=0.99 (p<0.05)<br>• Correlation between CA and BA (Pearson's correlation coefficient (r)=0.507 (p<0.05)<br>• GPA overestimated CA (MD=2 mos, p>0.05)<br>• GPA overestimated CA (MD=5.32 mos, p<0.05)<br>• GPA overestimated CA (MD=7 mos, p<0.05)<br>**GPA-Female**<br>• Intra-observePearson'son' correlation coefficient (r) = 0.99 (p<0.05)<br>• Inter-observer (Pearson's correlation coefficient (r) = 0.99 (p<0.05)<br>• Correlation between CA and BPearson'son' correlation coefficient (r) = 0.461, p<0.05)<br>**MT - Male**<br>• Intra-observerPearson'sn' correlation coefficient (r)=0.97 (p<0.05)<br>• Inter-observer (Pearson's correlation coefficient (r)=0.97 (p<0.05)<br>• Correlation between CA and BA (Pearson's correlation coefficients (r)=0.454, p<0.05)<br>• MT underestimated CA (MD=13.57 mos, p=0.000)<br>**MT - Female**<br>• Intra-observer Pearson' correlation coefficients = 0.94 (p<0.05)<br>• Inter-observer Pearson' correlation coefficients = 0.97 (p<0.05)<br>• MT underestimated CA (MD=20.57 mos, p = 0.000)
Schmidt *et al*. (2007) (54,67)	Observational Retrospective cohort	**Accuracy**	N=649 left hand/wrist radiograph from German subjects aged 1 to 18 yrs<br>Orthopedic practice, Papenburg, Germany.	**Skeletal method**<br>*GPA*<br>Thiemann and Nitz's radiographic atlas	**GPA**<br>**GPA-Males**<br>• Differences between CA and BA (MD = −0.49 yrs, SD=2.02; p < 0.05)<br>• Correlation between CA and BA (Determination coefficient (r^2) = 0.930, p<0.05)<br>**GPA-Females**<br>• Differences between CA and BA (MD = −0.39 yrs, SD=2.16, p < 0.05)

					• Correlation between CA and BA (Determination coefficient (r^2) = 0.922 ($p<0.05$)<br>**Thiemann Nitz method - Males**<br>• Differences between CA and BA (MD = −0.05 yrs, SD=1.65, $p<0.05$)<br>• Correlation between CA and BA (Determination coefficient (r^2) = 0.955 ($p < 0.05$)<br>**Thiemann Nitz method - Females**<br>• Differences between CA and BA (MD = +0.05 yrs, SD=1.74, $p < 0.05$)<br>• Correlation between CA and BA (Determination coefficient (r^2) = 0.927 ($p<0.05$)
Soudack *et al.* (2012) 55	Observational Retrospective cohort	**Accuracy**	N = 679 children n = 375 men (55.22%), n = 304 (44.77%) women from Israel who underwent left hand/wrist x-rays.<br>Edmond and Lily Safra Children's Hospital, Chaim Sheba Medical Center, Tel Hashomer, Israel.	**Skeletal method**<br>*GPA*	**GPA**<br>**GPA - Male**<br>• Inter-rater agreement (Cohen`s kappa coefficient) = 0.371 (p=0.0177)<br>• Intraclass correlation coefficient (ICC) = 0.9846<br>• Differences between CA and BA (MD = 0.23 yrs, $p < 0.05$)<br>**GPA - Female**<br>• Inter-rater agreement (Cohen`s kappa coefficient) = 0.4667 (p=0.005)<br>• Intraclass correlation coefficient (ICC)= 0.9787<br>• Differences between CA and BA (MD = 0.10 yrs, $p < 0.05$)<br>**Overall group by time**<br>***T1***=1-6 yrs old.<br>• Differences between CA and BA (MD= 2.3 mos, $p<0.0001$.<br>***T2***= 6-10 yrs old.<br>• Differences between CA and BA MD=5.4 mos, $p<0.0001$)<br>***T3***= 10 -15 yrs old.<br>• Differences between CA and BA (MD= 3.7 mos, $p<0.0001$)<br>T4= 15 -18 yrs old.<br>• Differences between CA and BA (MD=−2.9 mos, $p<0.0043$)
Tineo *et al.* (2006) (81)	Observational Prospective cohort	**Accuracy**	N= 30 (n=15, 46.7% females, n=16, 53.3% males left hand/wrist radiograph from Venezuelan children aged 6 to 12 yrs<br>Facultad de Odontología de La Universidad del Zulia, Maracaibo, Zulia, Venezuela.	**Skeletal method**<br>*GPA*<br>**Dental method**<br>*DM* (Demirjian's classification system)	**GPA**<br>• Correlation between CA and BA (Pearson correlation coefficient (r) = 0.918 ($p<0.05$)<br>**DM (Demirjian's classification system)**<br>• Correlation between CA and DA (Pearson correlation coefficient (r)= 0,929 ($p<0.05$)
Tiwari *et al.* (2020) (39)	Observational Prospective cohort	**Accuracy**	N = 70 children and adolescents aged 1 to 19 yrs n=37 males (52.86%), n=33 females (47.14%) from the population of eastern Uttar Pradesh underwent left hand/wrist x-rays.	**Skeletal method**<br>*GPA*	**GPA**<br>• Differences between CA and BA (MD= 0.56 mos, SD= 1.33 yrs, p=0.001)<br>**GPA - Male**<br>• Differences between CA and BA (MD=9.03 mos, SE= 0.25, $p \leq 0.05$)<br>**GPA - Female**<br>• Differences between CA and BA (MD=4.33 mos, SE = 0.18, $p \leq 0.05$)

			Institute of Medical Sciences, Varanasi, India.		**Overall group by time**<br>***T1***=0-5 yrs old.<br>• Differences between CA and BA (MD=0.89 yrs, SD= 0.85 yrs, p = 0.03)<br>***T2***=10-15 yrs old.<br>• Differences between CA and BA (MD=0.81 yrs, SD= 1.57 yrs, p = 0.03).
Tsehay *et al.* (2017) (75)	ObservationCross-sectionalnal study	**Accuracy**	N = 108 children and adolescents aged 10 to 22 yrs n=65 males (60.19%), n=43 females (39.81%) left hand/wrist x-rays from Debre Markos, East Gojjam, Ethiopia.	**Skeletal method**<br>*GPA*	**GPA**<br>**GPA - Male**<br>• Correlation between CA and BA (r = 0.912, p = .000).<br>• GPA overestimated CA (MD=8.7 mos, p<0.05).<br>**GPA - Female**<br>• Correlation between CA and BA (r=0.761, p =.000).<br>• GPA overestimated CA (MD=11.8 mos, p<0.05).
Van Rijn *et al.* (2001) (68)	Observational Retrospective cohort	**Accuracy**	N=572 children and adolescents aged 5 to 19.9 n=278 males (48.60%), n=294 females (51.40%) left hand/wrist x-rays from Dutch Caucasians.<br>University Hospital Rotterdam, Rotterdam, The Netherlands.	**Skeletal method**<br>*GPA*	**GPA**<br>• Intra-observer coefficient = 2.4 %<br>**GPA-Male**<br>• Correlation between CA and BA (r = 0.979, p<0.001)<br>**GPA-Female**<br>• Correlation between CA and BA (r = 0.974, p<0.001)
Wenzel *et al.* (1984) (70)	Observational Prospective cohort	**Accuracy**	N= 637 Austrian children and adolescents aged 7 to 16 n=459 males (72.06%), n=178 females (27.94%) left hand/wrist x-rays from Graz.<br>Institute of Radiology, Royal Dental College, Aarhus, Denmark	**Skeletal method**<br>*GPA*<br>*TW2*<br>*TW 20-bone*	**GPA - Male**<br>• Differences between CA and BA (p < 0.01)<br>**GPA-Female**<br>• Differences between CA and BA (p=0.4 < p< p=0.5)<br>**TW2-Male**<br>• Differences between CA and BA (p < 0.01)<br>**TW2-Female**<br>• Differences between CA and BA (p=0.1 < p< p=0.2)<br>**TW 20-bone**<br>• Differences between CA and BA (p=0.7 < p< p=0.8)
Zabet *et al.* (2015) (65)	Observational Retrospective cohort	**Precision**<br>*Reproducibility*<br>**Accuracy**	N=190 children n=100 males (52.63%), n=90 females (47.37%) aged 10 to 19 yrs from France underwent left hand/wrist x-rays.<br>Department of Radiology, University Hospital of Tours, France.	**Skeletal method**<br>*GPA*	**GPA**<br>• Inter-rater agreement (Cohen`s kappa coefficient) = 0.96 (p=0.0177)<br>• Differences between CA and BA (MD= −2.29 mos, SD=10, p<0.05)<br>**GPA-Male**<br>• Correlation between CA and BA (r = 0.98 (p< 0.05)<br>• Correlation between CA and BA (Determination coefficient (r^2) = 0.84 (p<0.01)<br>**GPA-Female**<br>• Correlation between CA and BA (r) = 0.93 (p<0.05)<br>• Correlation between CA and BA (Pearson's determination coefficient (r^2) = 0.74 (p<0.01)
Zafar *et al.* (2010) (45)	Observational	**Accuracy**	N = 889 children [n = 535 males (60.18%), n = 354 females (39.82%)]	**Skeletal method**<br>*GPA*	**GPA**

diagnostics

MDPI

	Cross-sectional study	aged up to 216 mos from a tertiary care hospital in Karachi underwent left hand/wrist radiographs. Karachi, Pakistan.	• Correlation between CA and BA (Pearson correlation coefficient (r)= 0.992 (p<0.001)<br>• Differences between CA and BA (MD=0.4 mos, p=0.584).

Abbreviations: BA: bone age; CA: chronological age; DA: dental age; DM: Demirjian's classification system; EO Ebrí-carpal (EOIC); metacarpophalangic (EOIMF); Carpometacarpophalangic (EOICMF); GPA: Greulich–Pyle Atlas; KS: Korean Standard Bone age chart; MD: mean difference; MK: MacKay's method, MT: Maturos method; OPG: orthopantomography; RUS–CHN: China 05; TW: Tanner–Whitehouse–Healey; TW2-RUS: Tanner–Whitehouse-2 radius, ulna, and short bone; TW3: Tanner–Whitehouse 3; VA: vertebral age.
